# Author Correction: Plasma-assisted manipulation of vanadia nanoclusters for efficient selective catalytic reduction of NO_x_

**DOI:** 10.1038/s41467-024-51375-w

**Published:** 2024-08-13

**Authors:** Yong Yin, Bingcheng Luo, Kezhi Li, Benjamin M. Moskowitz, Bar Mosevitzky Lis, Israel E. Wachs, Minghui Zhu, Ye Sun, Tianle Zhu, Xiang Li

**Affiliations:** 1 School of Space and Environment, Beihang University, Beijing, 100191 China; 2 College of Science, China Agricultural University, Beijing, 100083 China; 3 Institute of Engineering Technology, Sinopec Catalyst Co. Ltd., Beijing, 101111 China; 4 Operando Molecular Spectroscopy & Catalysis Laboratory, Department of Chemical and Biomolecular Engineering, Lehigh University, Bethlehem, PA 18015 USA; 5 State Key Laboratory of Chemical Engineering, East China University of Science and Technology, 130 Meilong Road, Shanghai, 200237 China

**Keywords:** Heterogeneous catalysis, Pollution remediation, Catalyst synthesis

Correction to: *Nature Communications* 10.1038/s41467-024-47878-1, published online 27 April 2024

In this article the author’s name Bar Mosevitzky Lis was incorrectly written as Bar Mosevizky Lis. The original article has been corrected.

