# Supplementary material for: Plasma-assisted manipulation of vanadia nanoclusters for efficient selective catalytic reduction of NOx
Source: Nat Commun. 2024 Apr 27;15:3592. doi: 10.1038/s41467-024-47878-1 (PMC11055856; doi:10.1038/s41467-024-47878-1)
Supplement: Supplementary file 1 — Supplementary Information [file 41467_2024_47878_MOESM1_ESM.pdf]

Supplementary Information

Plasma-Assisted Manipulation of Vanadia Nanoclusters for Efficient Selective Catalytic Reduction of NO_x

Yong Yin et al.

Table of Contents

1 Supplementary Catalyst Preparation and Characterization 3

2 Supplementary Measurement of NH_3-SCR Activity and Kinetics 4

3 Ex Situ Characterization 6

4 NH_3-SCR Activity 15

5 Effect of Plasma Treatment on Catalyst 20

6 Exploration of Intermediate Surface Species and Active Sites under Reaction Conditions 24

7 Comparison of Observed Vibrational Modes and Their Assignments During MES of NH_3-SCR over VO_x-WO_x/TiO_2 37

8 Investigation on Reaction Pathways and Rate-determining Steps 41

1 Supplementary Catalyst Preparation and Characterization

Preparation of plasma-treated catalysts at different stages： The catalyst was treated with the H_2 plasma at different preparation stages. The supported WO_3/TiO_2 catalyst was prepared with the above-mentioned impregnation method, then treated with an H_2 plasma, V was impregnated on the prepared material. The as-prepared catalyst is denoted as V_2O_5-WO_3(P)/TiO_2. In addition, TiO_2 was also treated with an H_2 plasma following the same preparation method as for the OR catalyst. The as-prepared catalyst is denoted as V_2O_5-WO_3/TiO_2(P).

Composition and structure characterization: Bulk V contents were determined with ICP-OES (Agilent 5110). The Brunauer–Emmett–Teller (BET) surface area was analysed on a physisorption instrument (Micromeritics, ASAP2020, USA). X-ray diffraction spectroscopy (XRD) was recorded using an X-ray diffractometer (Bruker, Bruker D8 Advance, Germany).

2 Supplementary Measurement of NH_3-SCR Activity and Kinetics

The activity tests are performed with 100 mg catalyst within 40–60 meshes in a fixed-bed quartz reactor. Before the test, all the catalysts are calcined at 500 °C for 180 min with a heating rate of 2 $°C \cdot min^{-1}$. The simulated flue gas consists of 350 ppm of NO, 350 ppm of NH_3, 5 % of O_2, balanced with Ar, with a gas hourly space velocity (GHSV) of 375,000 h^{-1}. The concentrations of NO, NO_2, NH_3, and N_2O were monitored by a Fourier-transformed infrared gas detector (ABB MBGAS-3000). The temperature ranged from 200 to 450 °C. The NO conversion, N_2 selectivity, and reaction rate(r) are calculated by the following equations:

$$NO_x\ Conversion\ (\%)=\frac{[NO_x]_{in}-[NO_x]_{out}}{[NO_x]_{in}}\times 100\% \quad (1)$$

$$N_2\ Selectivity\ (\%)=(1-\frac{2[N_2O]_{out}}{[NO_x]_{in}-[NO_x]_{out}+[NH_3]_{in}-[NH_3]_{out}})\times 100\% \quad (2)$$

$$r=\frac{Q_{NO}ln(1-NO_x\ Conversion)}{M_{NO}m_{cat}} \quad (3)$$

where $[NO_x]_{in}$ and $[NH_3]_{in}$ representing gaseous concentrations of NO_x and NH_3 in the inlet; $[N_2O]_{out}$, $[NO_x]_{out}$, and $[NH_3]_{out}$ represent gaseous concentrations of N_2O, NO_x, and NH_3 in the outlet. Q_{NO}, M_{NO}, and m_{cat} represent the NO mass flow in a fixed-bed reactor, NO conversion, NO molecular weight, and weight of the catalyst, respectively.

Specific activity is calculated by dividing the amount of NO molecules converted per second by the per m^2 of the catalyst surface at 200°C. It is calculated by the following equation:

$$specific\ activity=\frac{r}{S_{BET}} \quad (4)$$

where S_{BET} is the BET surface area, and r is the reaction rate.

Turnover frequencies (TOF) are calculated by dividing the amount of NO molecules converted per second at low NO conversion (＜15 %) by the per V atoms on the surface of catalysts. It is calculated by the following equation:

$$TOF=\frac{r}{n_v} \quad (5)$$

where n_v is the vanadium number on the catalyst surface, r is the reaction rate. n_v is calculated by the following equation:

$$n_v = \frac{NS_{BET}y}{N_A} \qquad (6)$$

where N, S_{BET}, y, and N_A are the concentration of surface atomic($10^{19}/\mathrm{m}^2$), BET surface area, surface vanadium concentration (tested by XPS), and the Avogadro constant [1-5].

3 Ex Situ Characterization

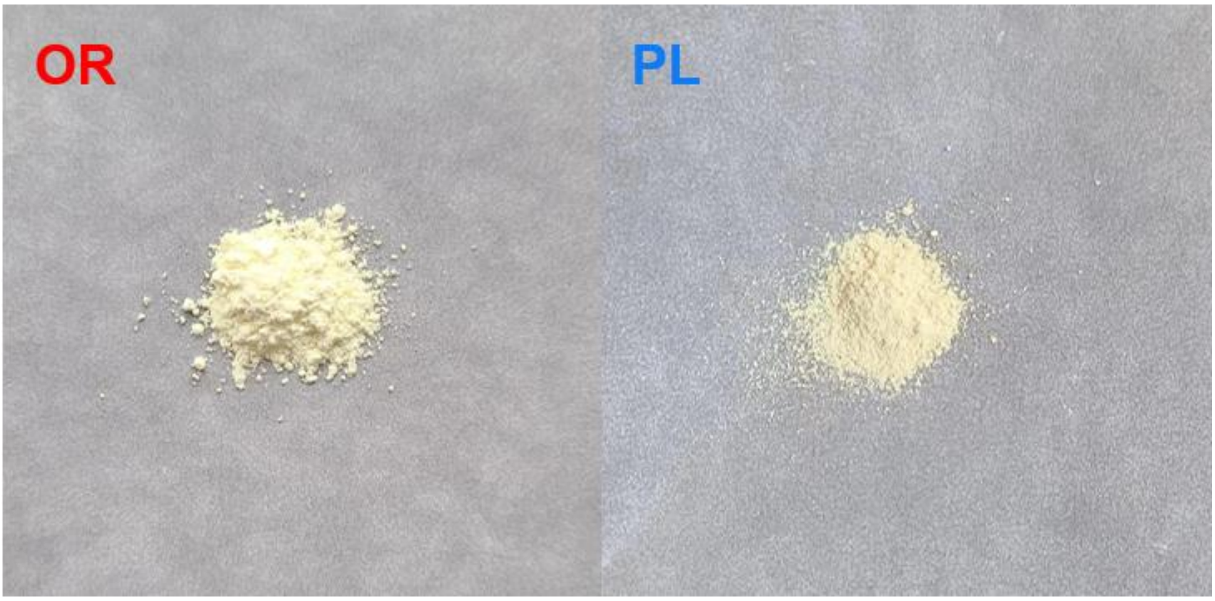

Figure S1 Sample photos of OR and PL catalysts.

Table S1 Elemental distribution characteristics, acidity, redox properties, and BET surface area of samples.

	Bulk V by ICP (wt%)	Surface V by XPS (wt%)	Bulk W by ICP (wt%)	Surface W by XPS (wt%)	H_2 consumption [a] (cm^3/g)	BET surface area (m^2/g)	Acid site amount [b] (μmol/g)
OR	0.81	0.91	3.16	4.05	3.2	63.9	57.7
PL	0.80	1.25	3.18	6.21	3.8	57.8	81.4

[a] test by H_2-TPR

[b] test by NH_3-TPD

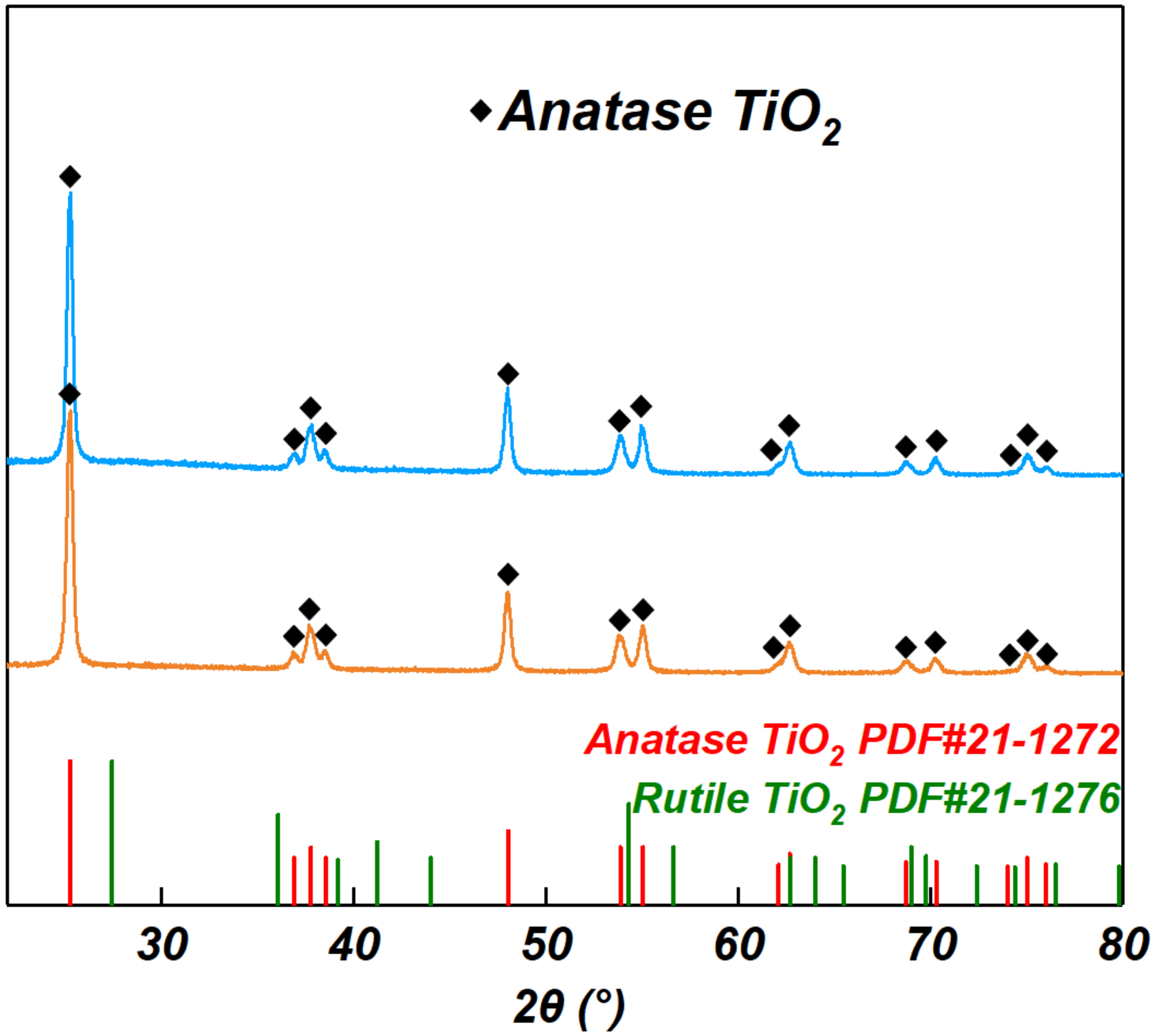

Figure S2 XRD profiles of OR (orange) and PL (blue).

Instruction: Both PL and OR exhibit well-defined diffraction peaks of rutile titanium dioxide.

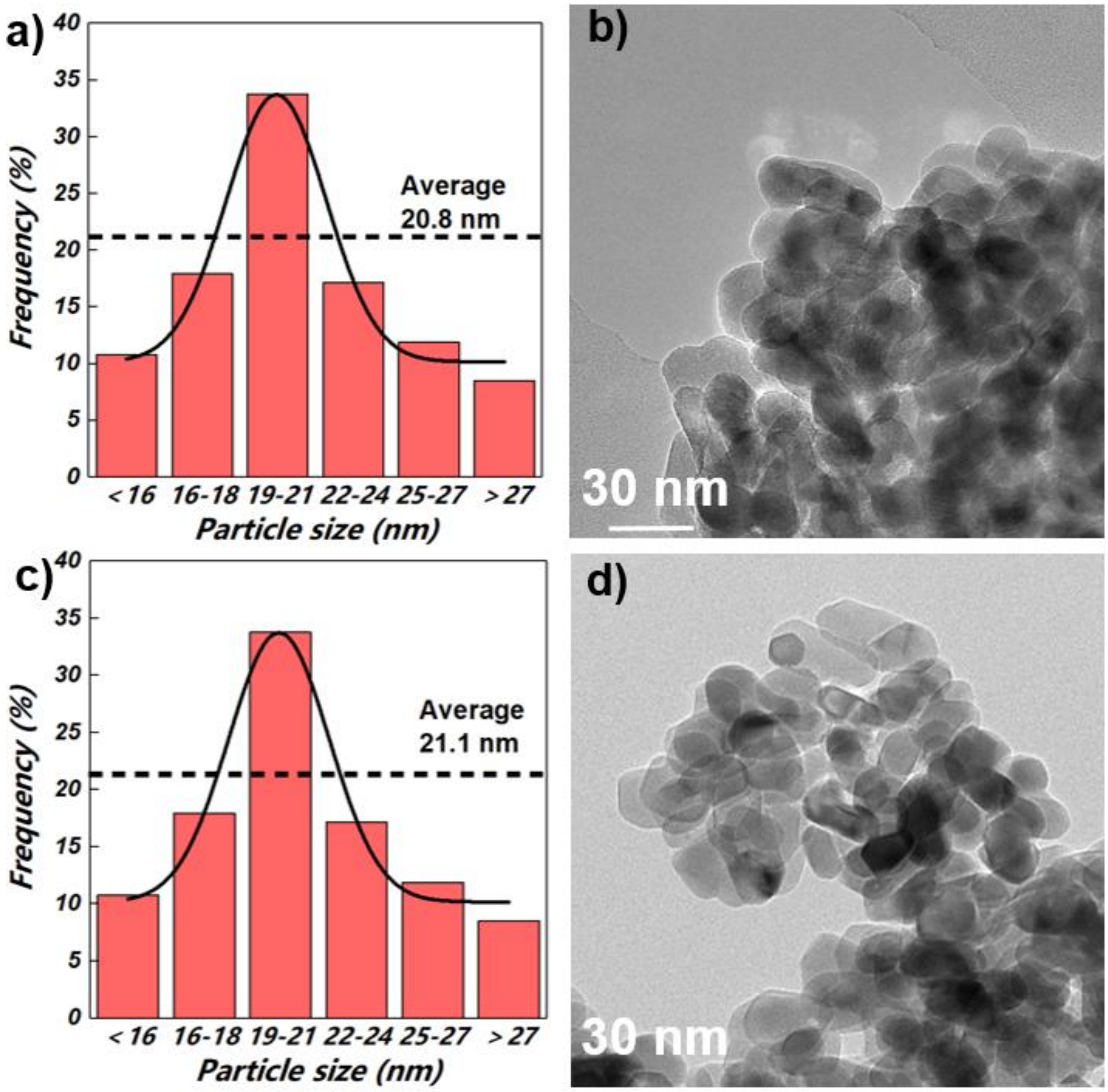

Figure S3 Particle size distributions of a) OR and c) PL. HRTEM images of b) OR and d) PL (90s) catalyst.

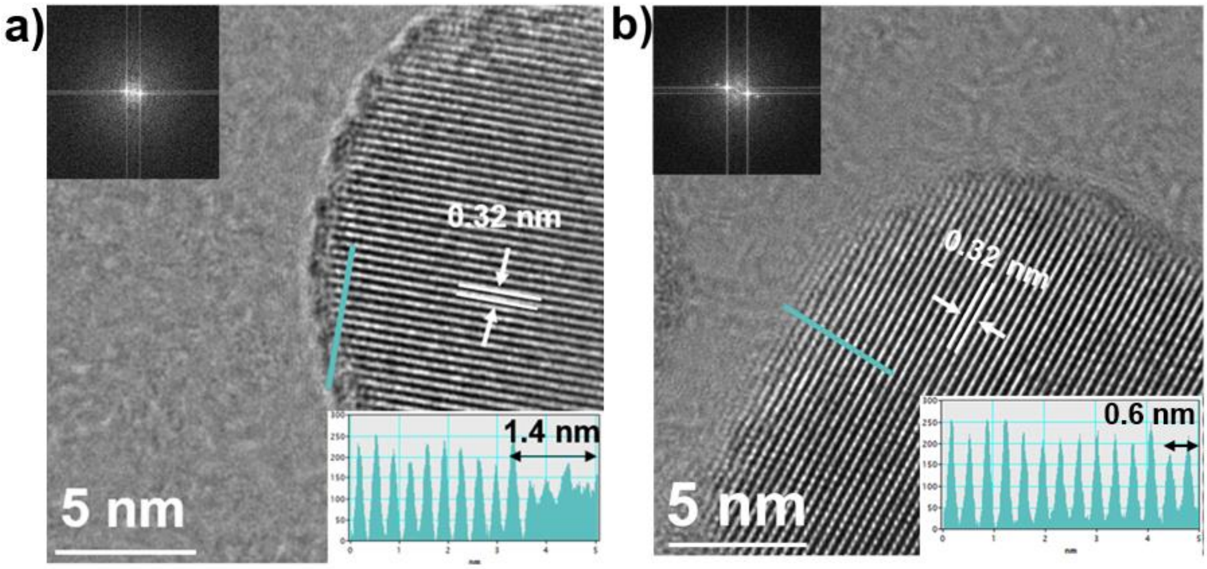

Figure S4 HRTEM images of PL (300s) a) and OR b). Insets: corresponding FFT images and image intensities of the selected areas.

Instruction: The roughness region at the edge of the catalyst after 300s of plasma treatment exhibits a thickness of 1.4 nm, which is higher than the 0.9 nm thickness recorded for the sample treated for 90s (PL).

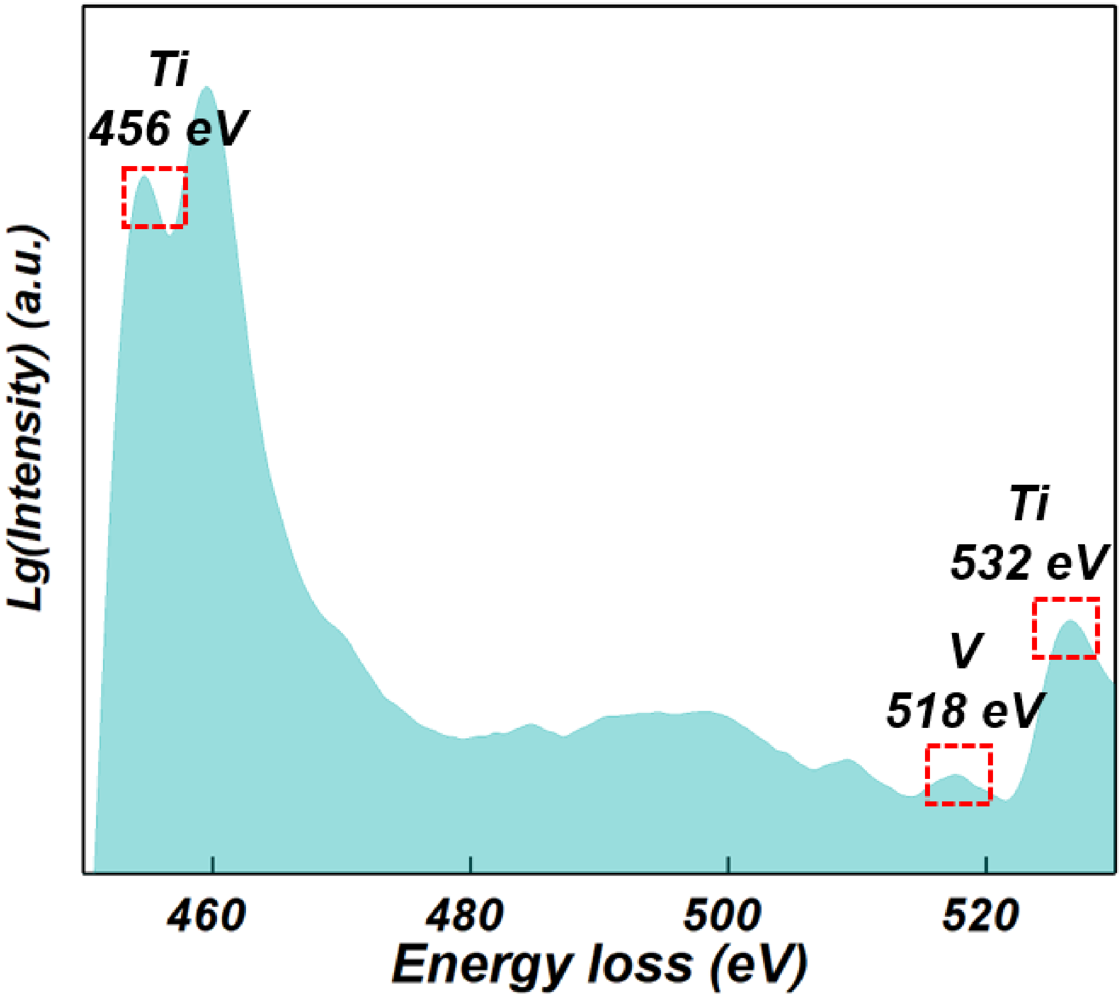

Figure S5 EELS spectrum of the red region in Fig 1g, the energy loss of titanium (Ti) is observed at 456 and 532 eV, while that of vanadium (V) is at 518 eV.

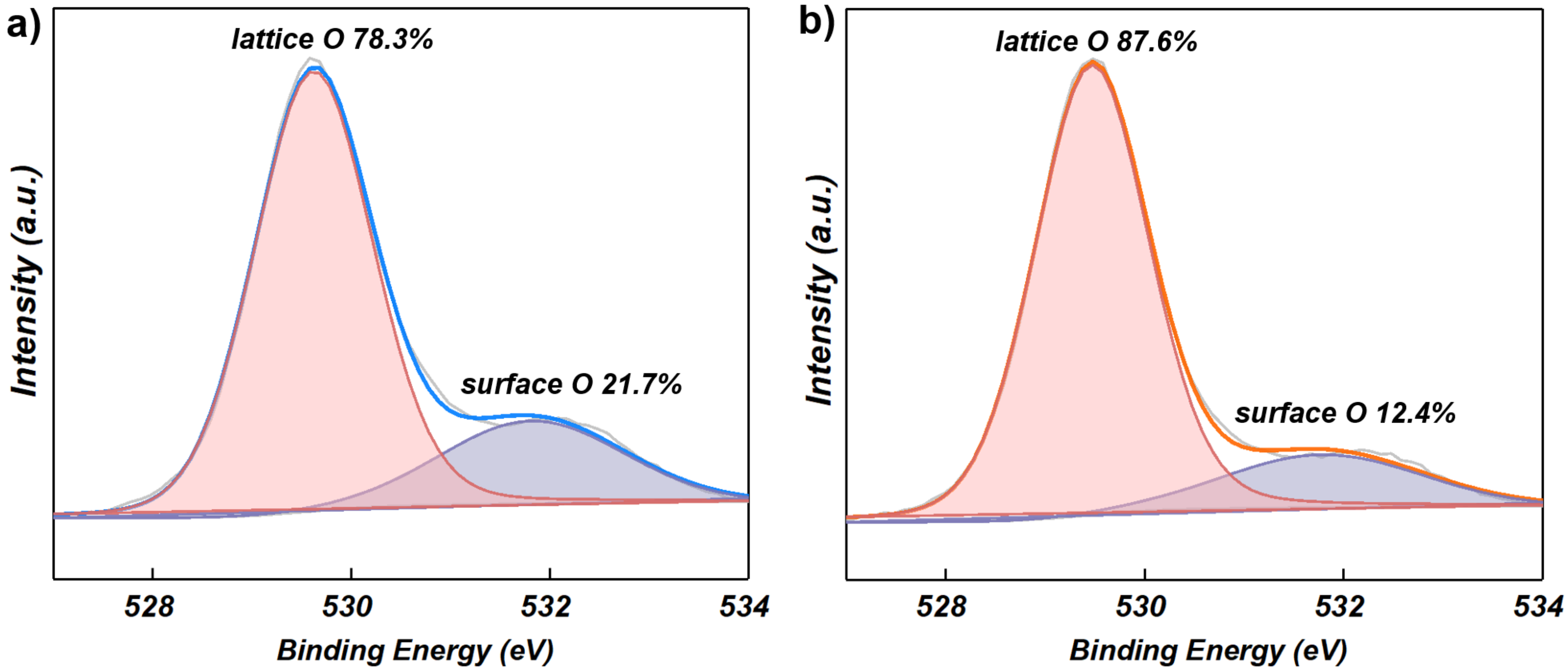

Figure S6 XPS O1*s* spectra of PL a) and PL b).

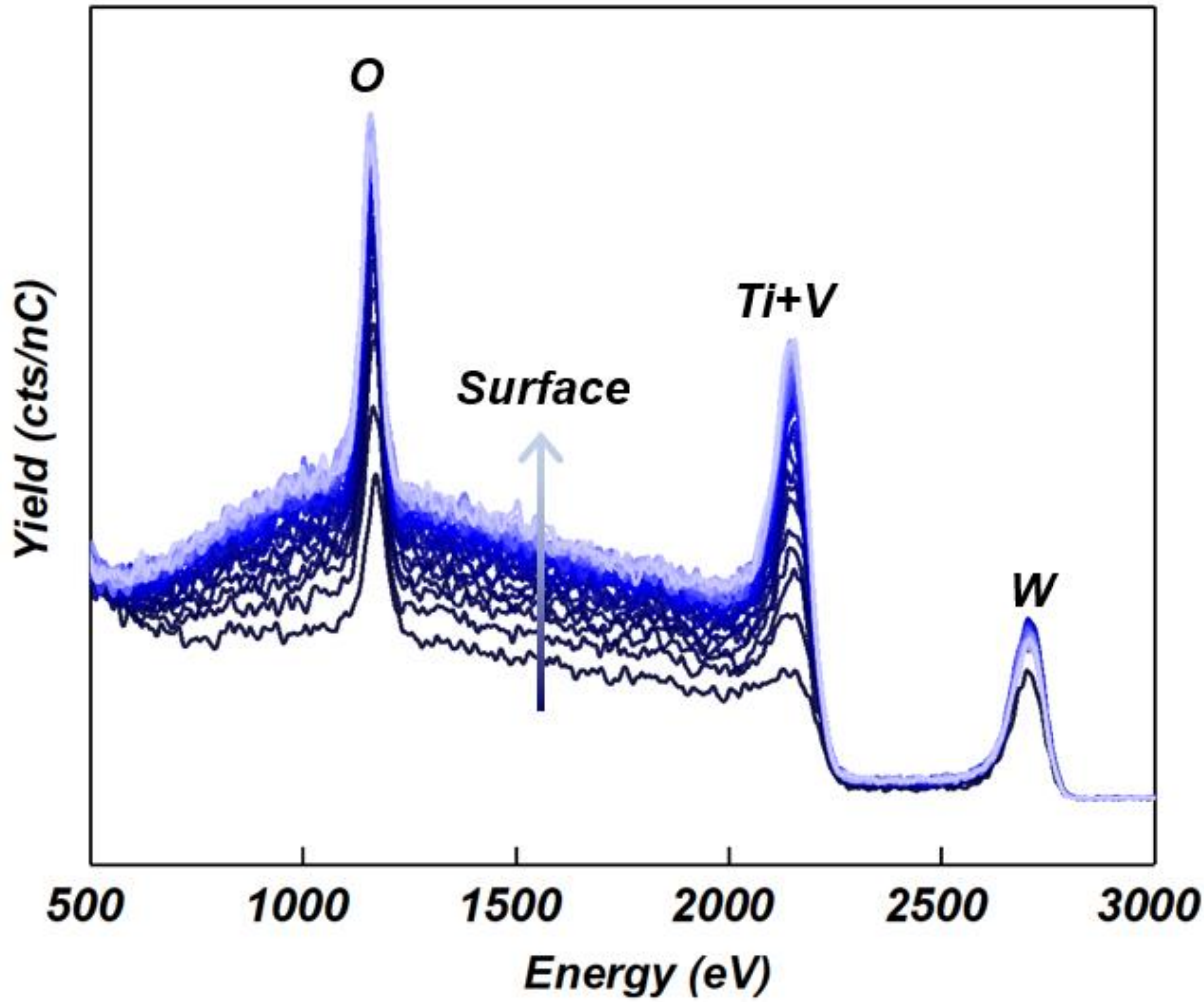

Figure S7 HS-LEIS spectral of PL, O. The peaks of O, Ti+V and W are located at 1155, 2152, and 2704 eV, respectively. The spectrum colour represents depths from the innermost to the topmost surface, with darker shades indicating deeper layers.

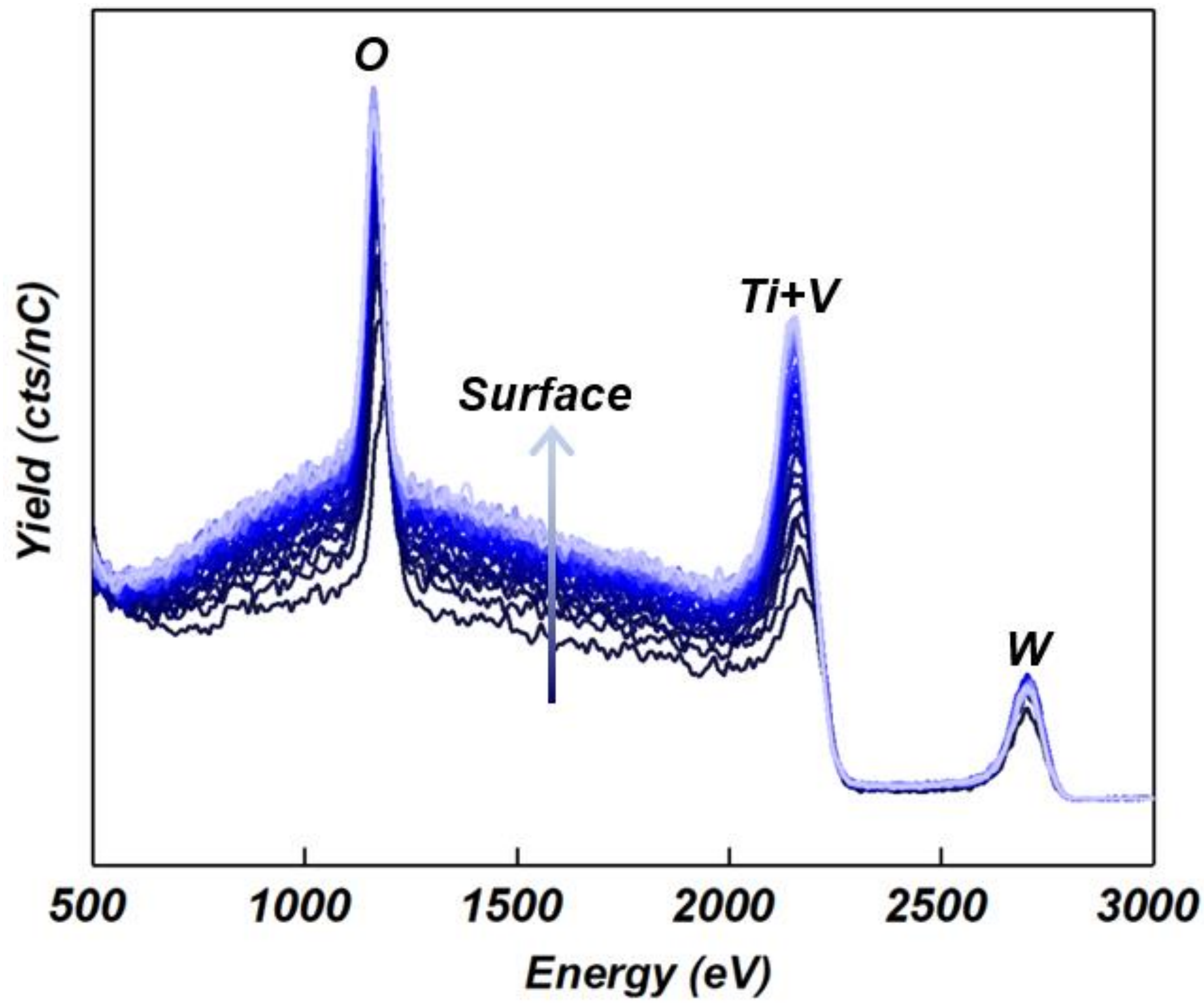

Figure S8 HS-LEIS spectral of OR. The peaks of O, Ti+V and W are located at 1155, 2152, and 2704 eV, respectively. The spectrum colour represents depths from the innermost to the topmost surface, with darker shades indicating deeper layers.

4 NH_3-SCR Activity

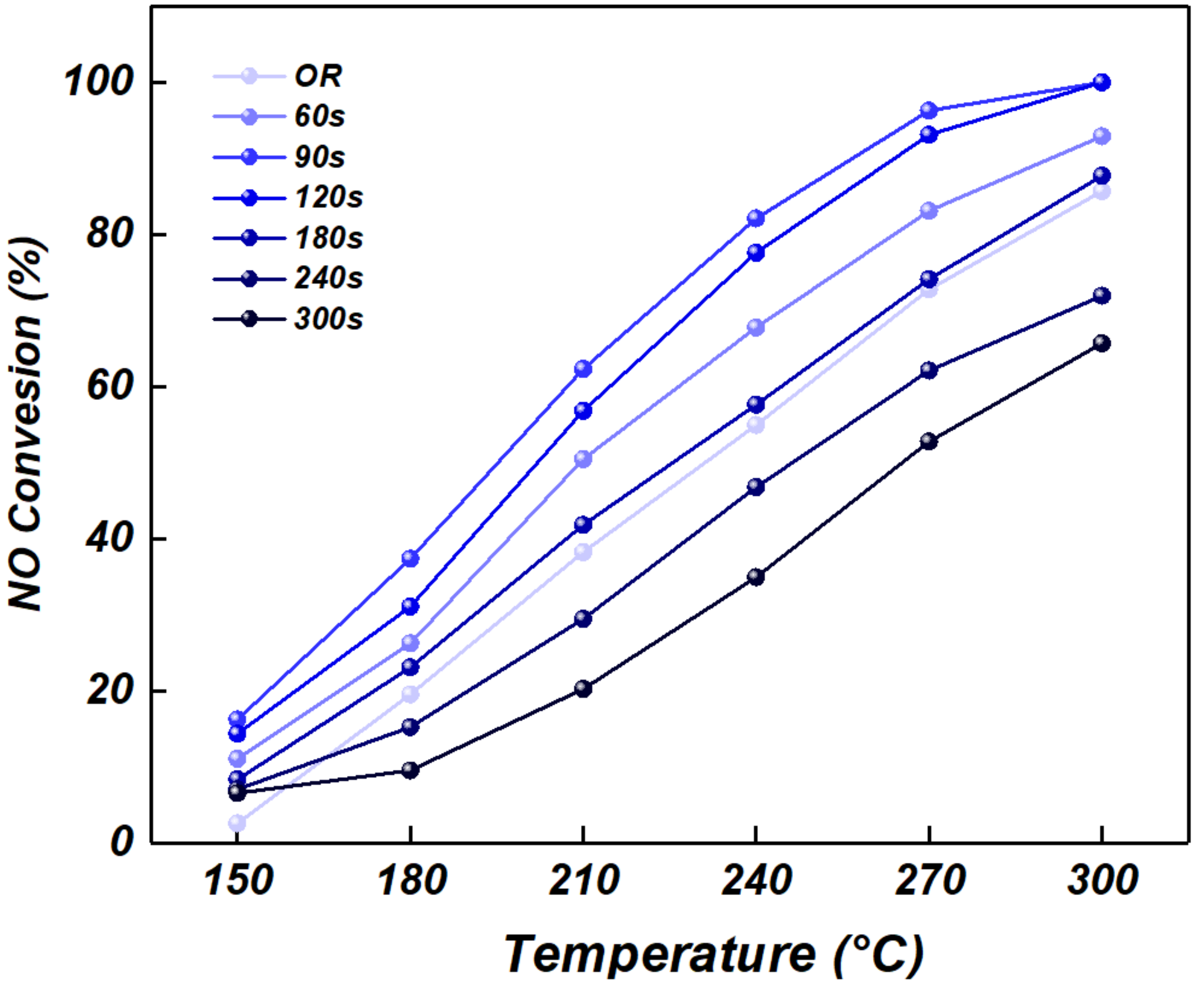

Figure S9 NO conversions of OR and PL with treatment time of 60, 90, 120, 180, 240 and 300s for NH_3-SCR.

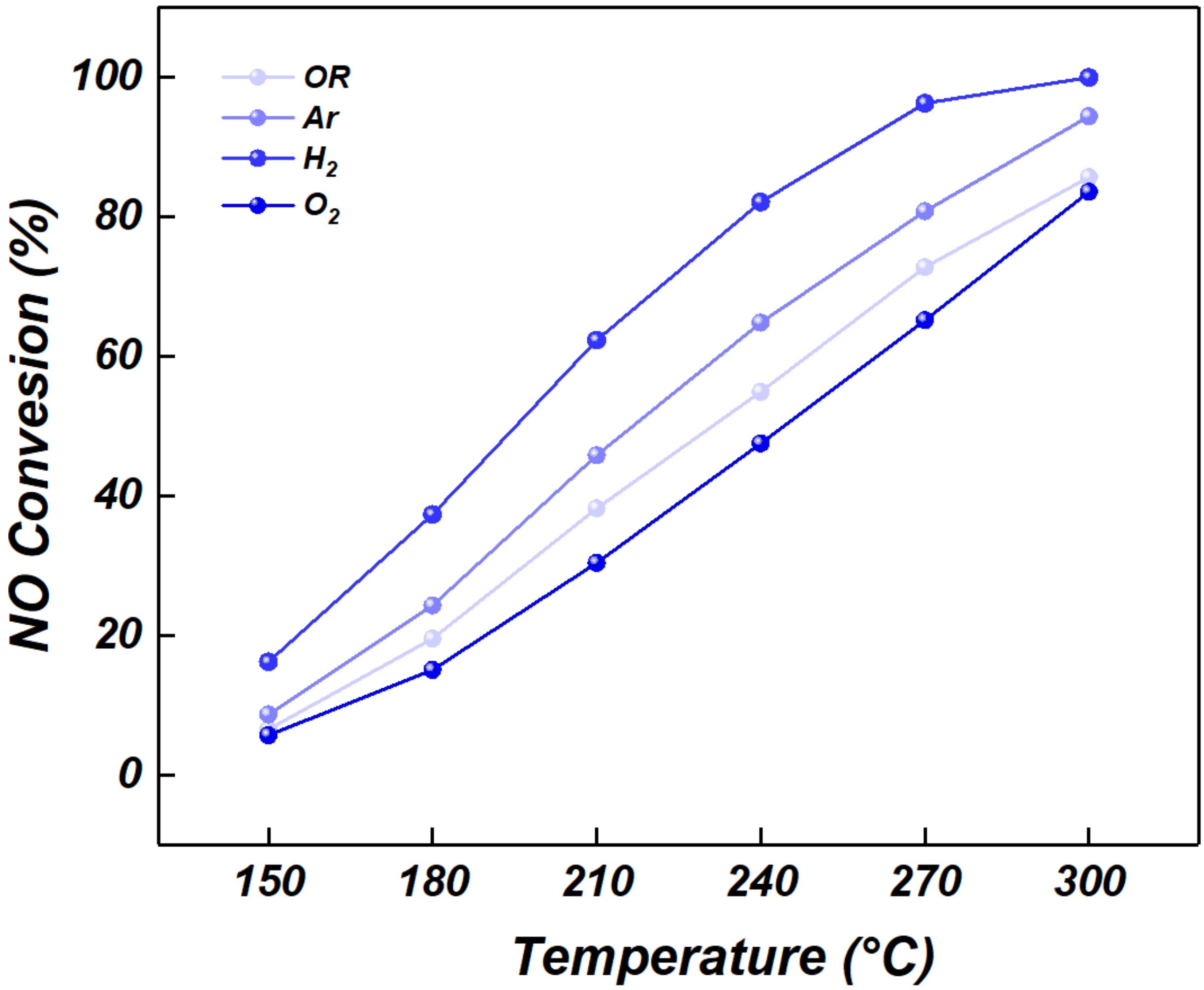

Figure S10 NO conversions of OR and PL with different treatment atmospheres (Ar, H_2, and O_2) for NH_3-SCR.

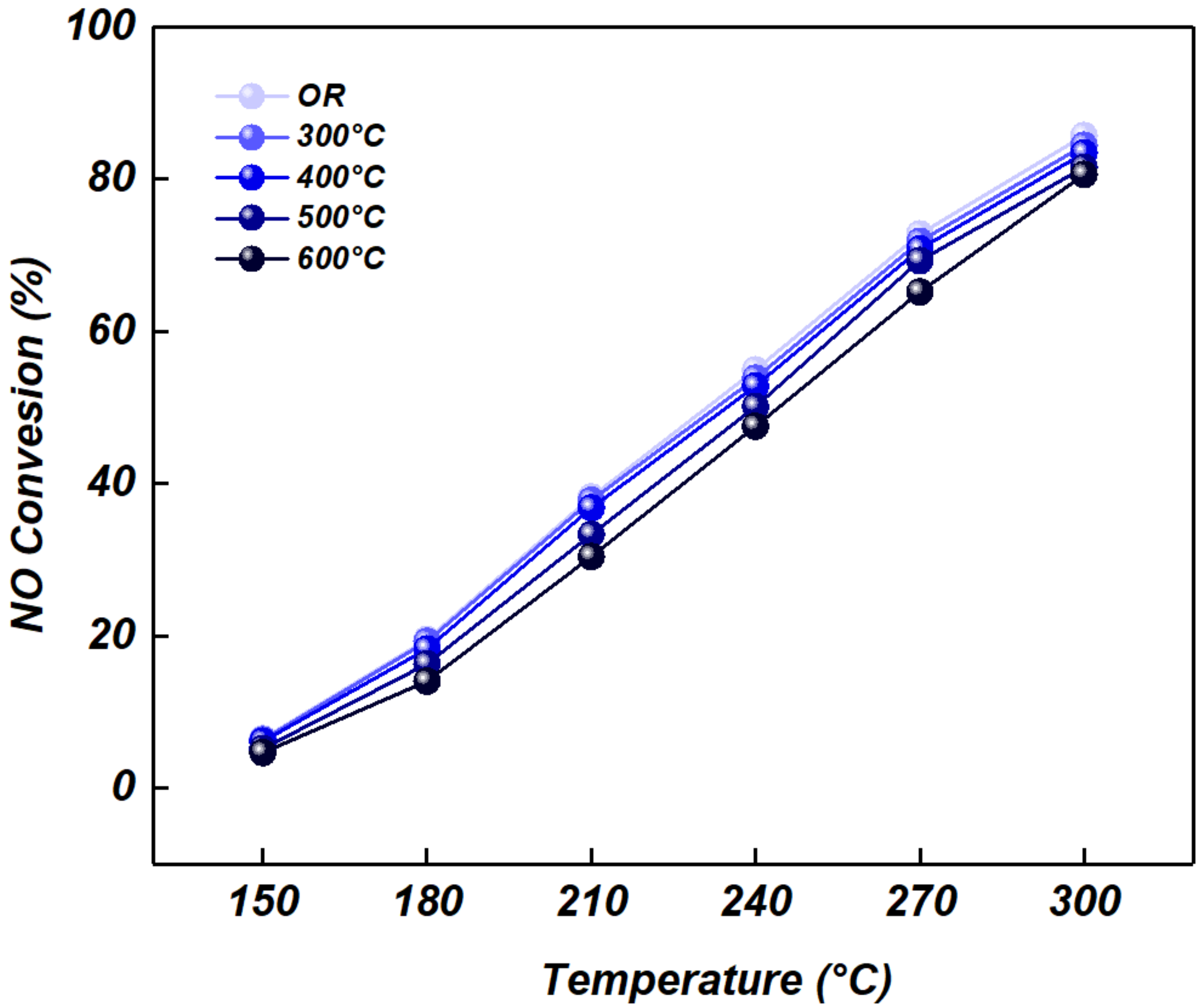

Figure S11 NO conversions of V_2O_5-WO_3/TiO_2 catalysts with H_2 thermal treatment control experiment at different temperatures (300 °C, 400 °C, 500 °C, and 600 °C) for NH_3-SCR.

Instruction: The catalytic activity of the catalyst decreased with increasing H_2 thermal treatment temperature, and a reduction of 20.1% was observed after treatment at 600℃. These results suggest that the thermal treatment did not enhance the catalytic activity of the material.

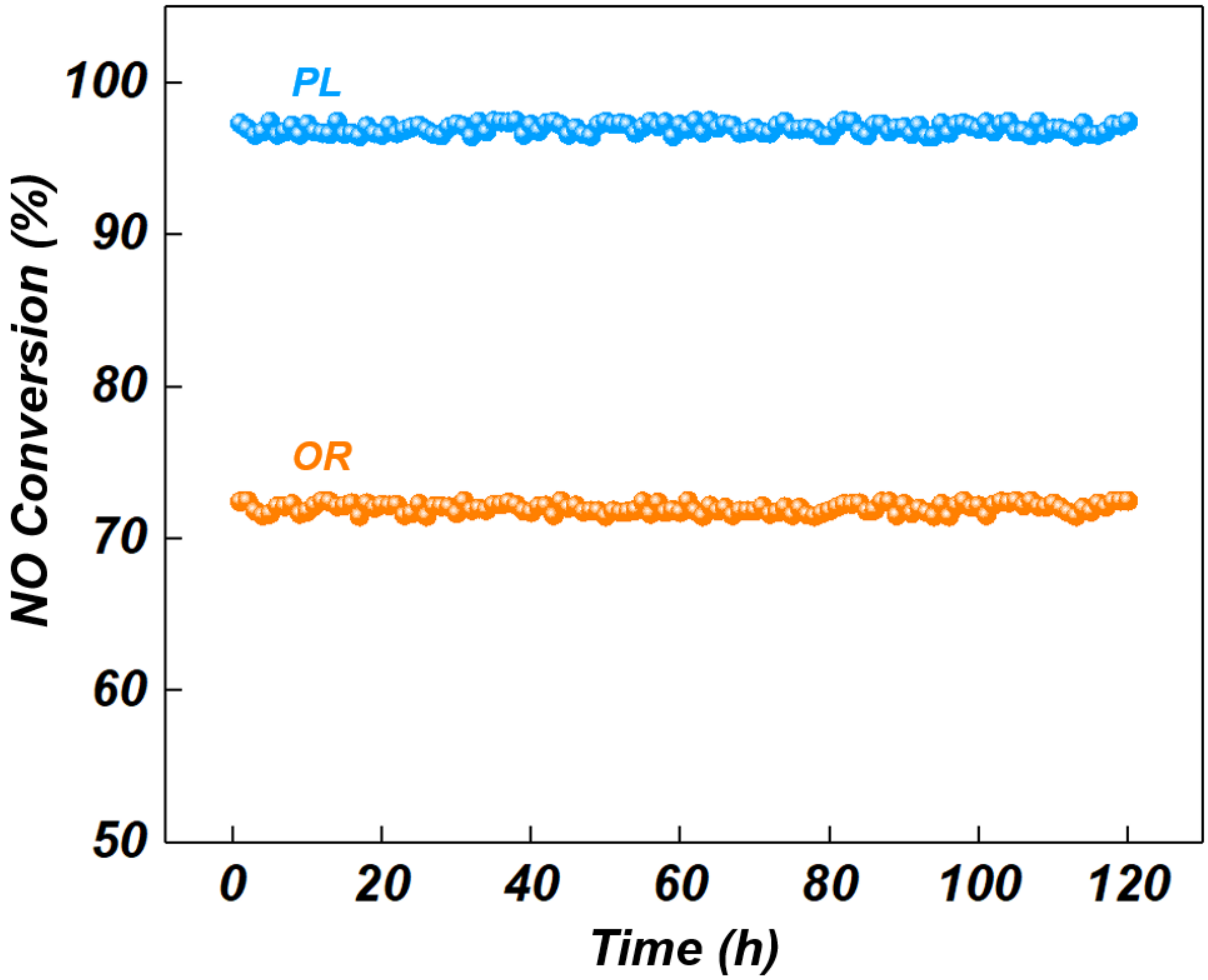

Figure S12 NO conversions of OR and PL at 270℃ during 120h running for NH_3-SCR.

Instruction: A 120-hour long-term stability test revealed that the NO conversion rates of PL and OR remained stable at 97±0.4% and 72±0.5%, respectively, suggesting that PL exhibits excellent catalytic stability.

Table S2 V, W and Mo content of different vanadia-based catalyst.

Catalyst	V content by ICP (wt%)		W content by ICP (wt%)		Mo content by ICP (wt%)	
	OR	PL	OR	PL	OR	PL
0.1VWTi	0.09	0.09	3.14	3.13	/	/
0.5VWTi	0.47	0.48	3.21	3.19	/	/
1VWTi	0.81	0.8	3.16	3.18	/	/
2VWTi	1.74	1.72	3.27	3.28	/	/
3VWTi	2.68	2.66	3.11	3.14	/	/
1VTi	0.83	0.83	/	/	/	/
1VMoTi	0.85	0.86	/	/	3.31	3.32

5 Effect of Plasma Treatment on Catalyst

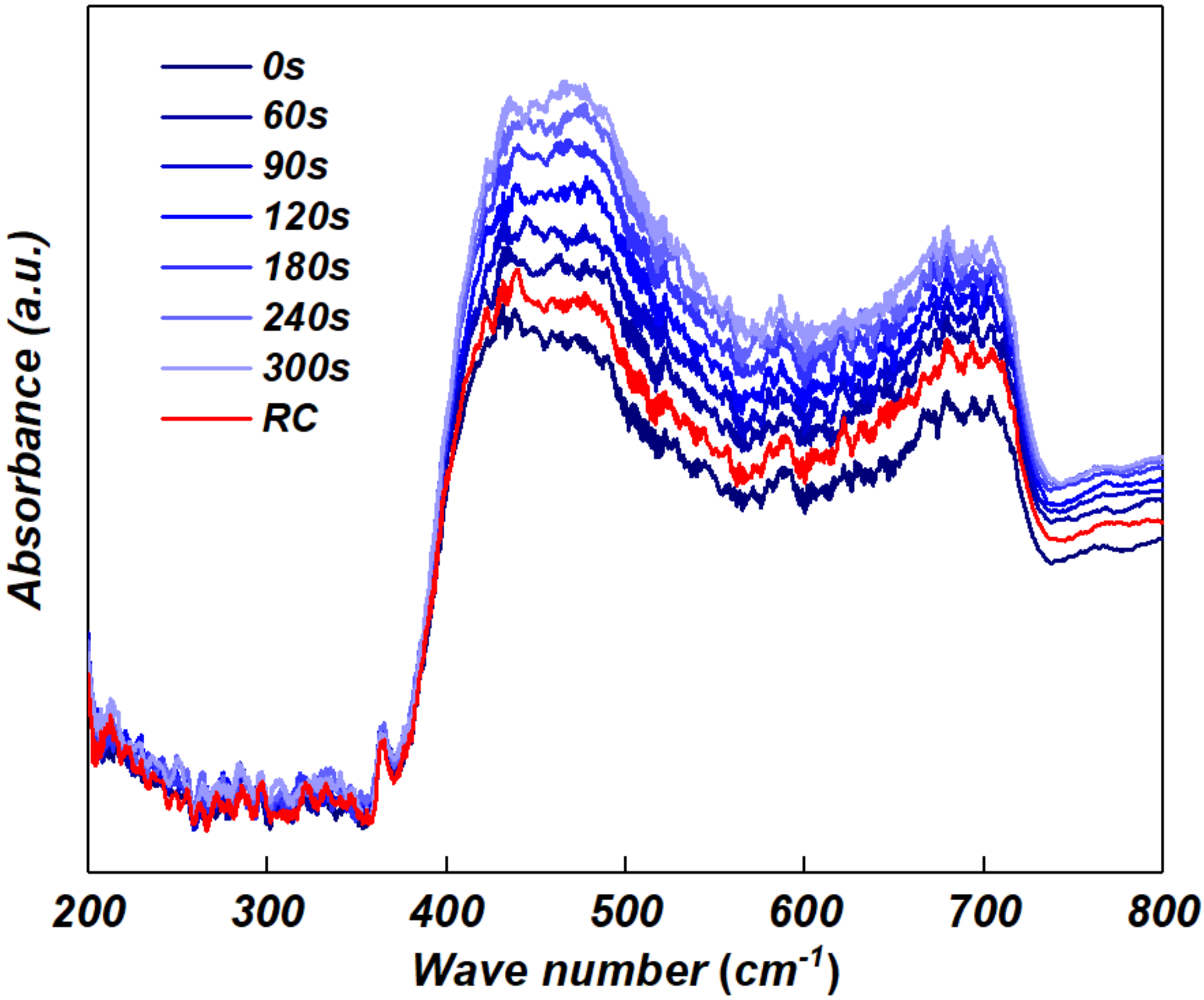

Figure S13 UV-vis spectra of TiO_2 with different plasma treatment time of 60, 90, 120, 180, 240 and 300s and re-calcined TiO_2 (RC).

Table S3 Edge energy values and sample photos of TiO_2 with different plasma treatment time and re-calcined TiO_2 (RC).

Treatment time/s	Eg/eV	Sample photos
0	3.21	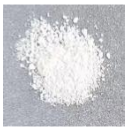
60	3.27	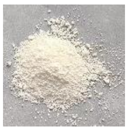
90	3.32	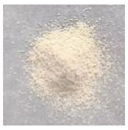
120	3.34	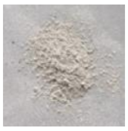
180	3.36	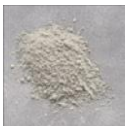
240	3.39	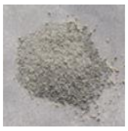
300	3.45	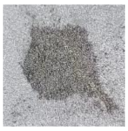
RC	3.25	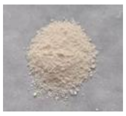

Instruction: The observed darkening of the colour and widening of the bandgap in titanium dioxide implies an increase in the number of defects in the material. The excess of defects potentially may affect the catalytic performance of the V_2O_5-WO_3/TiO_2 catalyst.

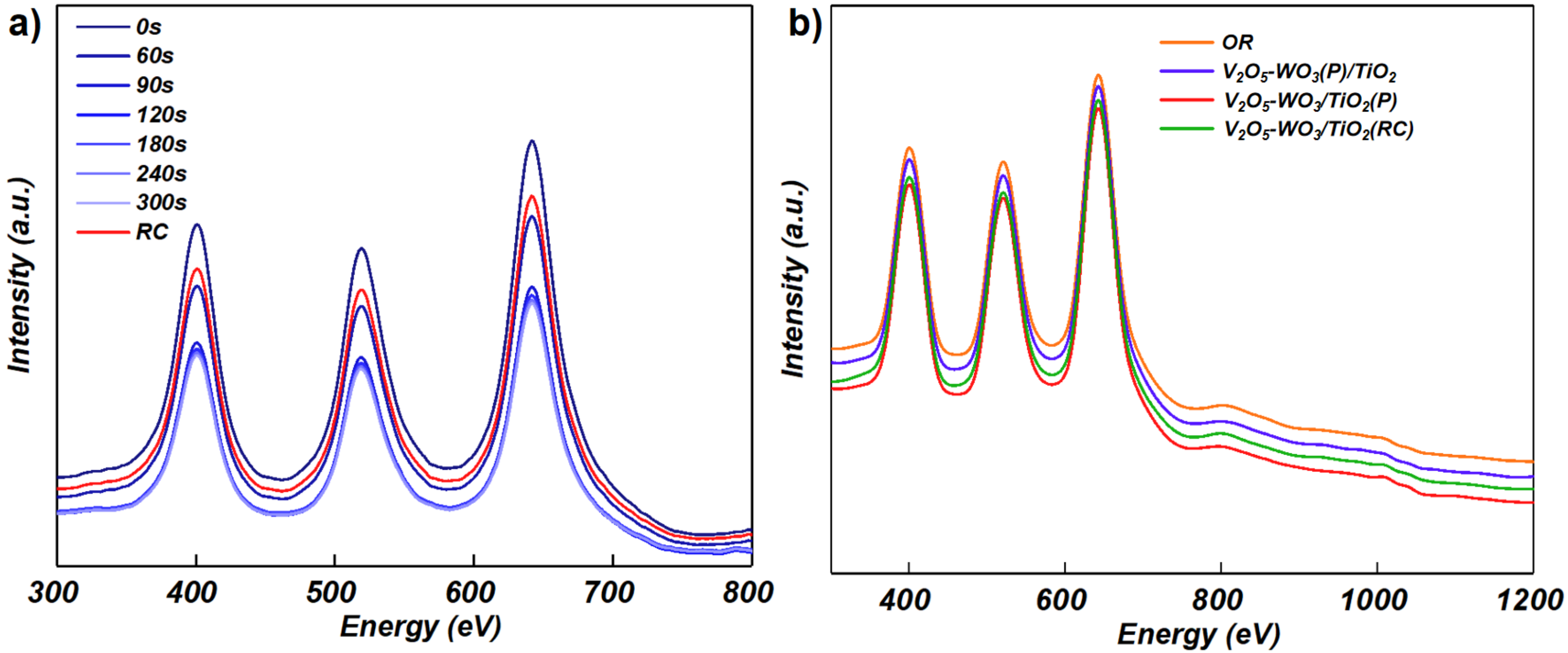

Figure S14 Raman spectra of a) TiO_2 with different plasma treatment time from 0-300s and Re-calcined TiO_2 (RC) and b) prepared V_2O_5-WO_3/TiO_2 and catalysts with different plasma treatment stage.

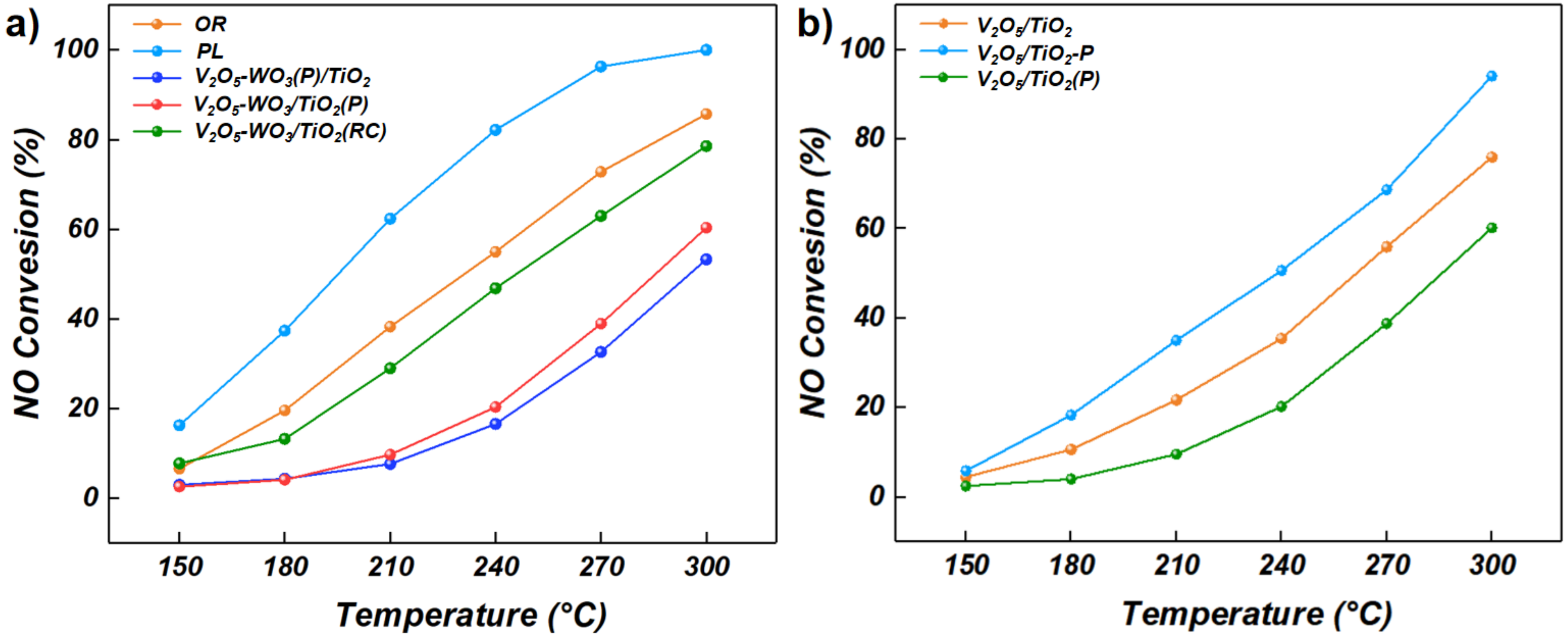

Figure S15 NO conversion of prepared a) V_2O_5-WO_3/TiO_2 and b) V_2O_5/TiO_2 catalysts with different plasma treatment stage for NH_3-SCR.

Clarification: The notation of (P) means plasma treatment stage. For instance, V_2O_5-WO_3(P)/TiO_2 represents V_2O_5 precursor impregnation on plasma treated WO_3/TiO_2, V_2O_5-WO_3/TiO_2(P) represents that V_2O_5 and WO_3 precursors simultaneous impregnation on plasma treated TiO_2, V_2O_5-WO_3/TiO_2(RC) represents that V_2O_5 and WO_3 precursors simultaneous impregnation on plasma treated and re-calcined TiO_2; V_2O_5/TiO_2-P: V_2O_5/TiO_2 plasma treated catalysts; V_2O_5/TiO_2(P): V_2O_5 precursor impregnation on plasma treated TiO_2.

6 Exploration of Intermediate Surface Species and Active Sites under Reaction Conditions

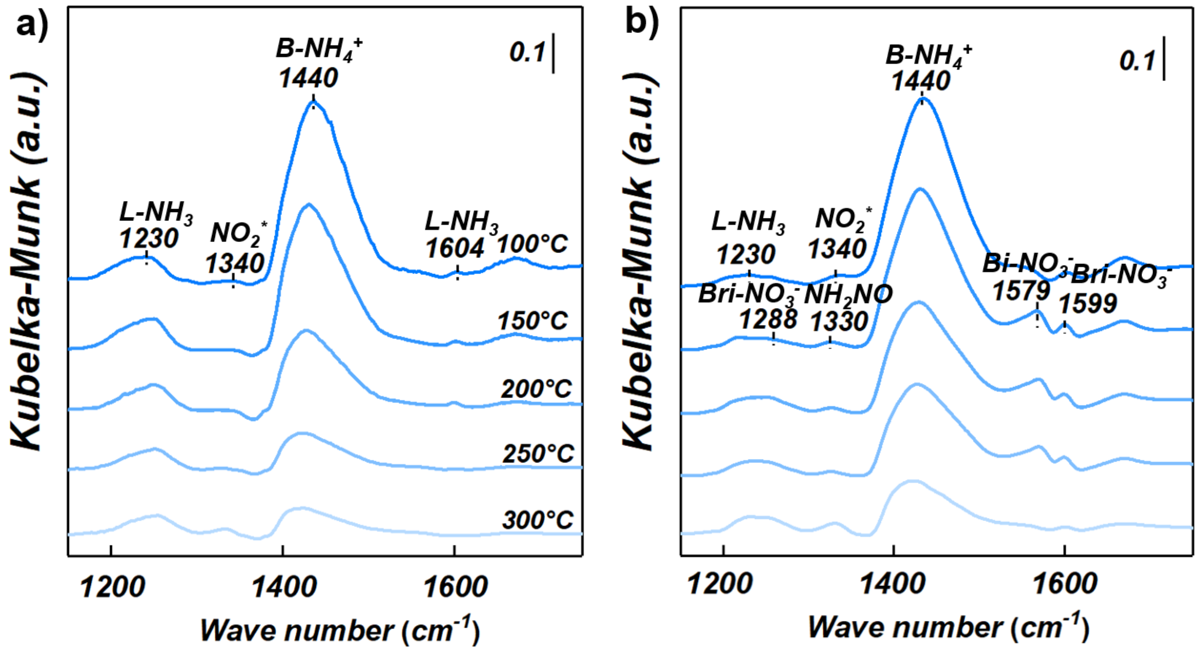

Figure S16 Time-resolved DRIFTS spectra of a) OR and b) PL with the rise in temperature from 100 °C for SCR. The sample was first treated in a 10 % O_2/Ar (50 ml/min) flow at 500 °C for 30 min. Then the reaction cell was cooled to 60 °C in an Ar flow (50 ml/min), the catalyst was heated from 60 to 500 °C at the heating rate of 10 °C/min and the spectra were collected as background spectra. Cooling the samples to the specified temperature, 2000 ppm of NH_3, 2000 ppm of NO, and 5 % O_2 (50 ml/min) were introduced into the *in situ* cell. The spectra were recorded as 32 scans at a resolution of 4 cm^{-1}.

Instruction: Beyond 150° C, the PL catalyst surface exhibits the formation of bridged nitrate (1288cm^{-1}, 1599cm^{-1}) and bidentate nitrate (1579 cm^{-1}) species, which persist up to 250 ° C.

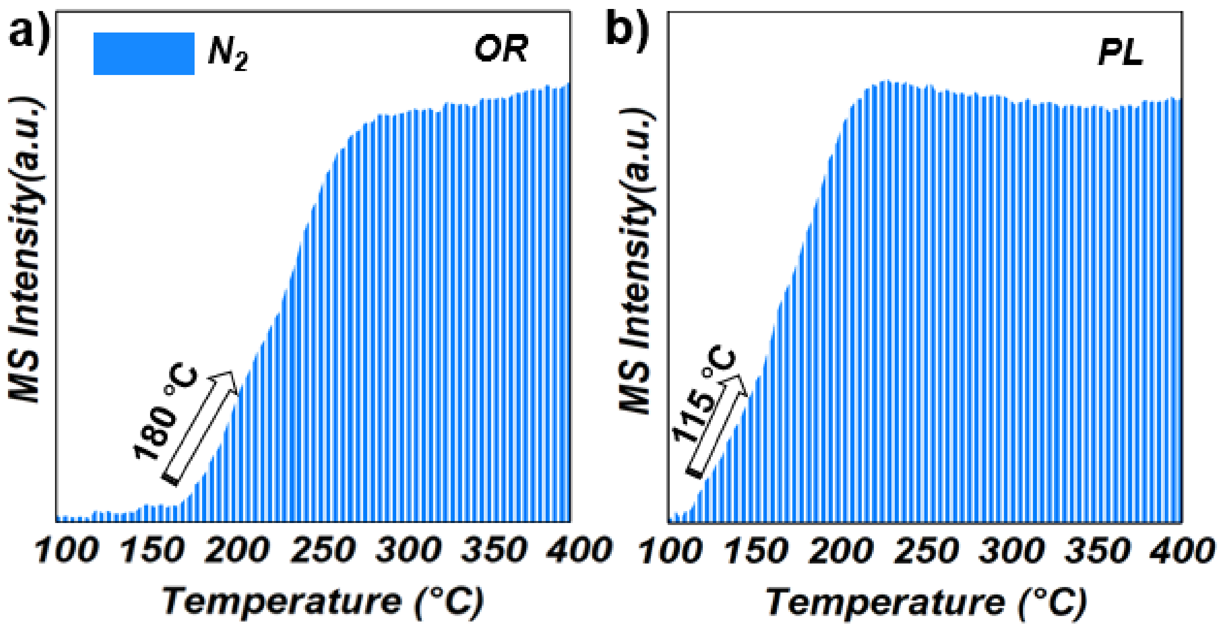

Figure S17 N_2 signal of a) OR and b) PL at the outset with the rise in temperature from 100℃ for SCR. An online mass spectrometer (MS, HIDEN mini HPR-20 R&D) tandem at the outlet of *in situ* cell.

Instruction: The observable N_2 production of the PL was 65℃ lower than that of OR, confirming its outstanding performance in the low-temperature NH_3-SCR.

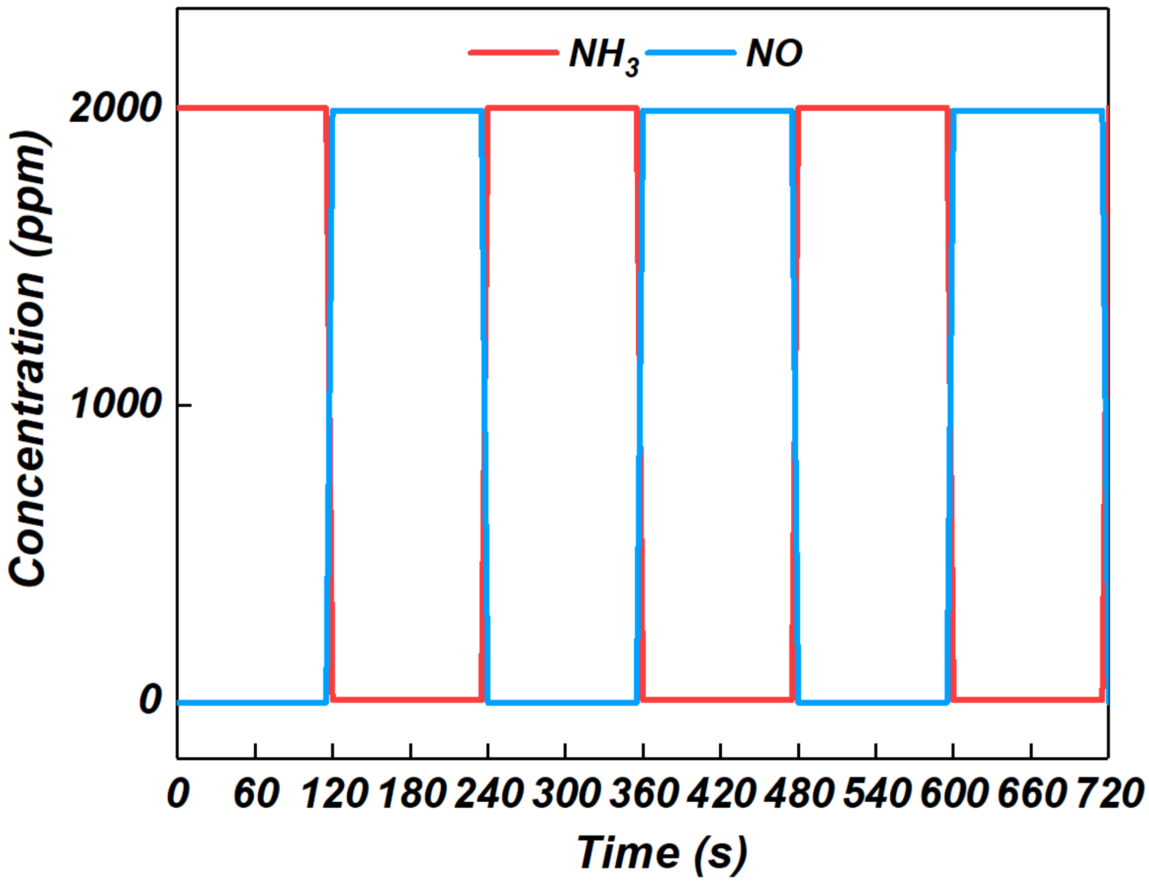

Figure S18 Representation of pulse sequence of NH_3 and NO during NO+O_2/NH_3+O_2 modulation experiment for MES experiment [6-8]. The exchange of NH_3 and NO atmospheres occurs at intervals of every 120 s, while the O_2 content remains unchanged.

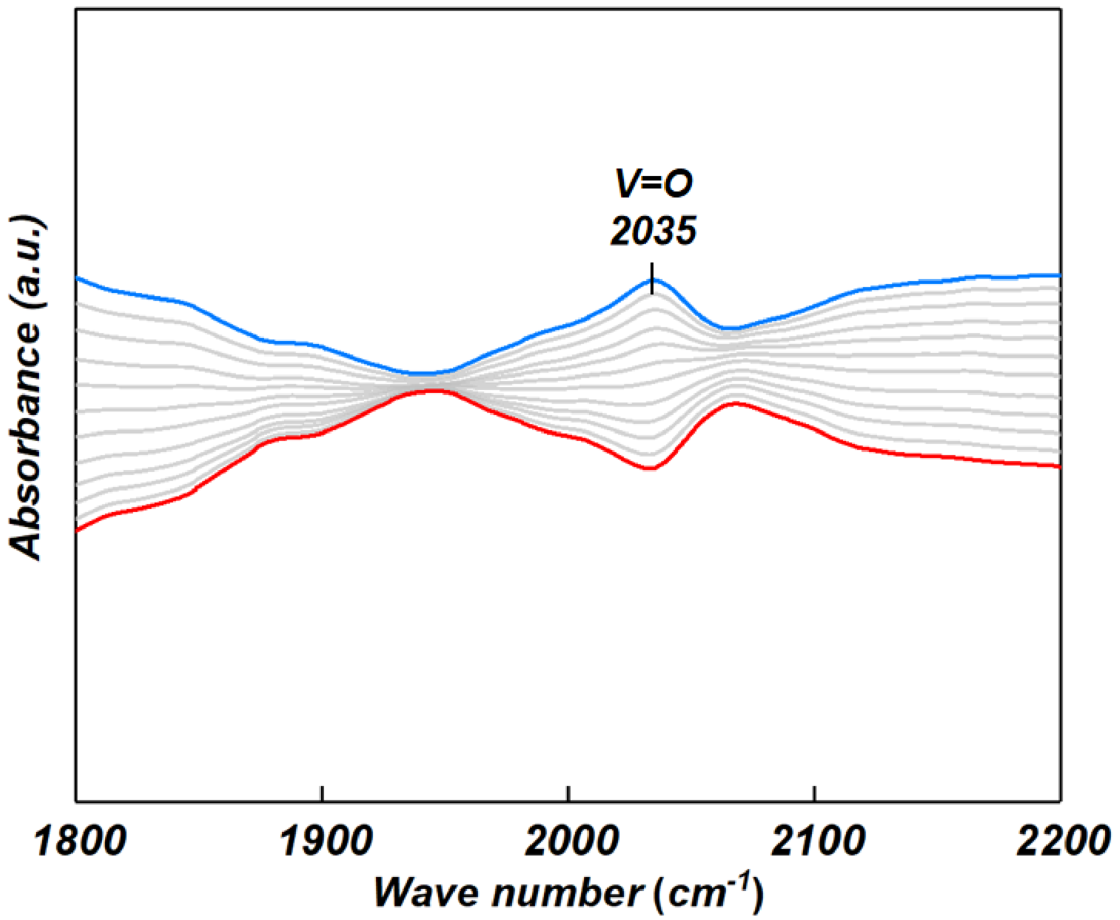

Figure S19 MES DRIFTS spectra of overtone region of PL catalyst during $NO+O_2/ND_3+O_2$ modulation experiment at 150°C. Red colour spectra represent the phase when NH_3 was introduced, while blue colour represents the phase when NO was introduced.

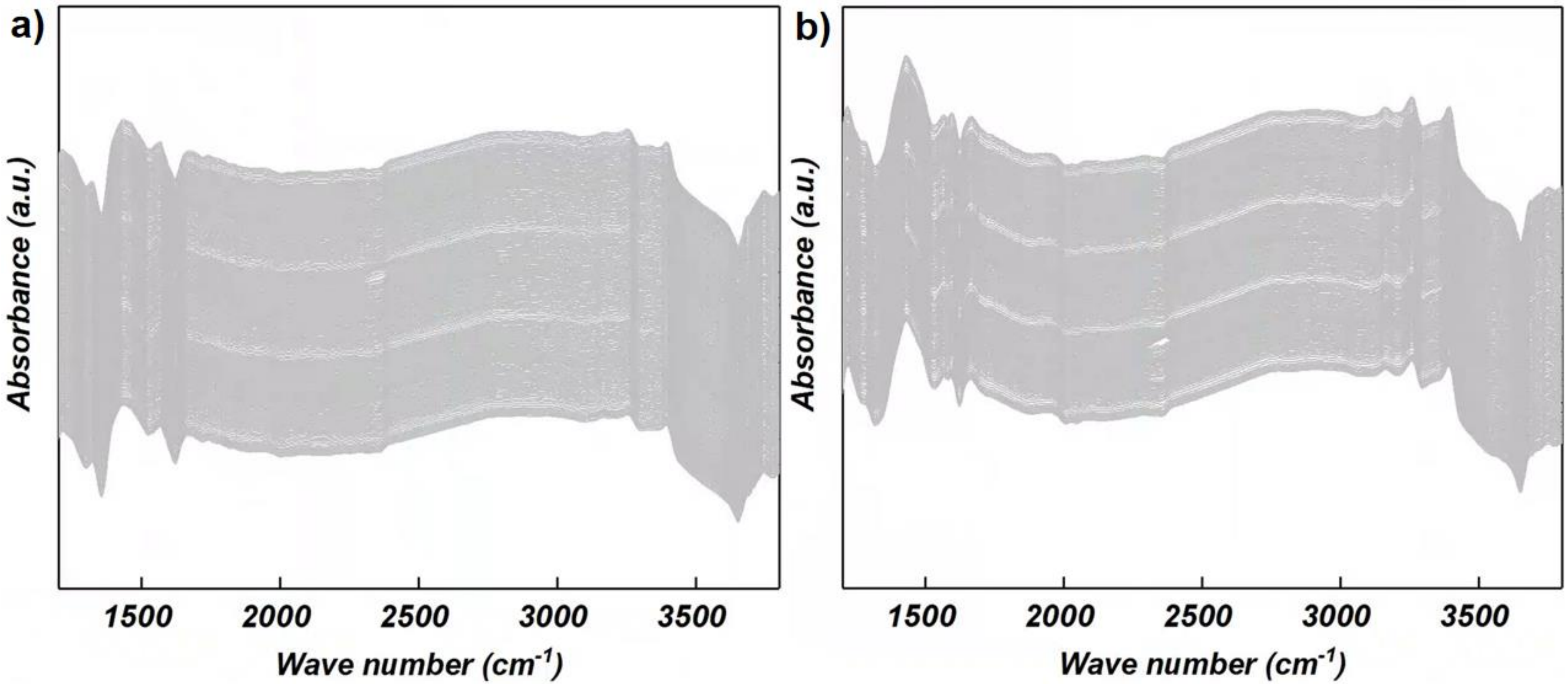

Figure S20 Time-resolved DRIFTS spectra at the wave number range of 1200-3800 cm^{-1} of a) OR and b) PL during $NO+O_2/NH_3+O_2$ modulation experiment at 150℃.

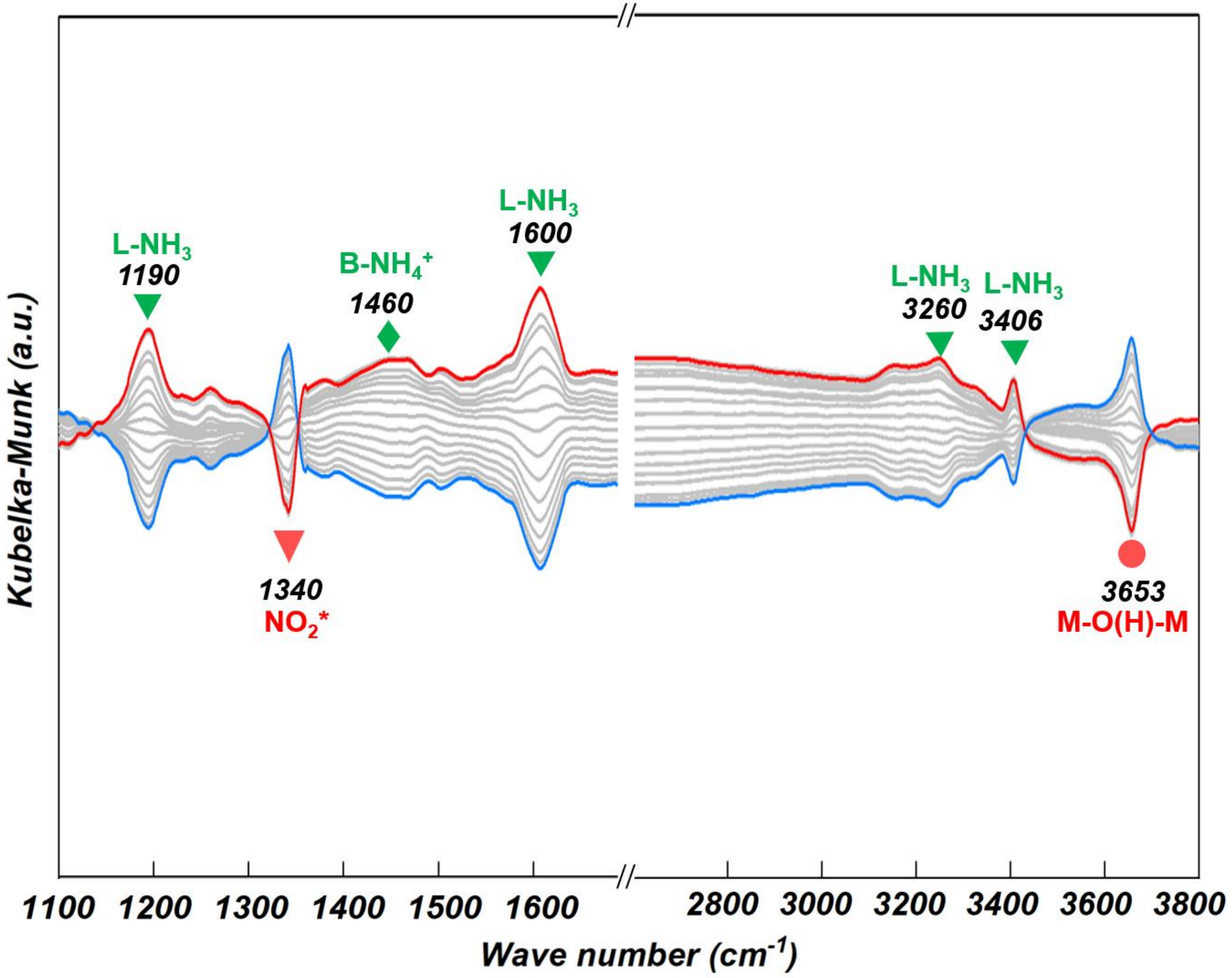

Figure S21 MES DRIFTS spectra of OR catalyst during $NO+O_2/NH_3+O_2$ modulation experiment. Red colour spectra represent the phase when NH_3 was introduced, while blue colour represents the phase when NO was introduced.

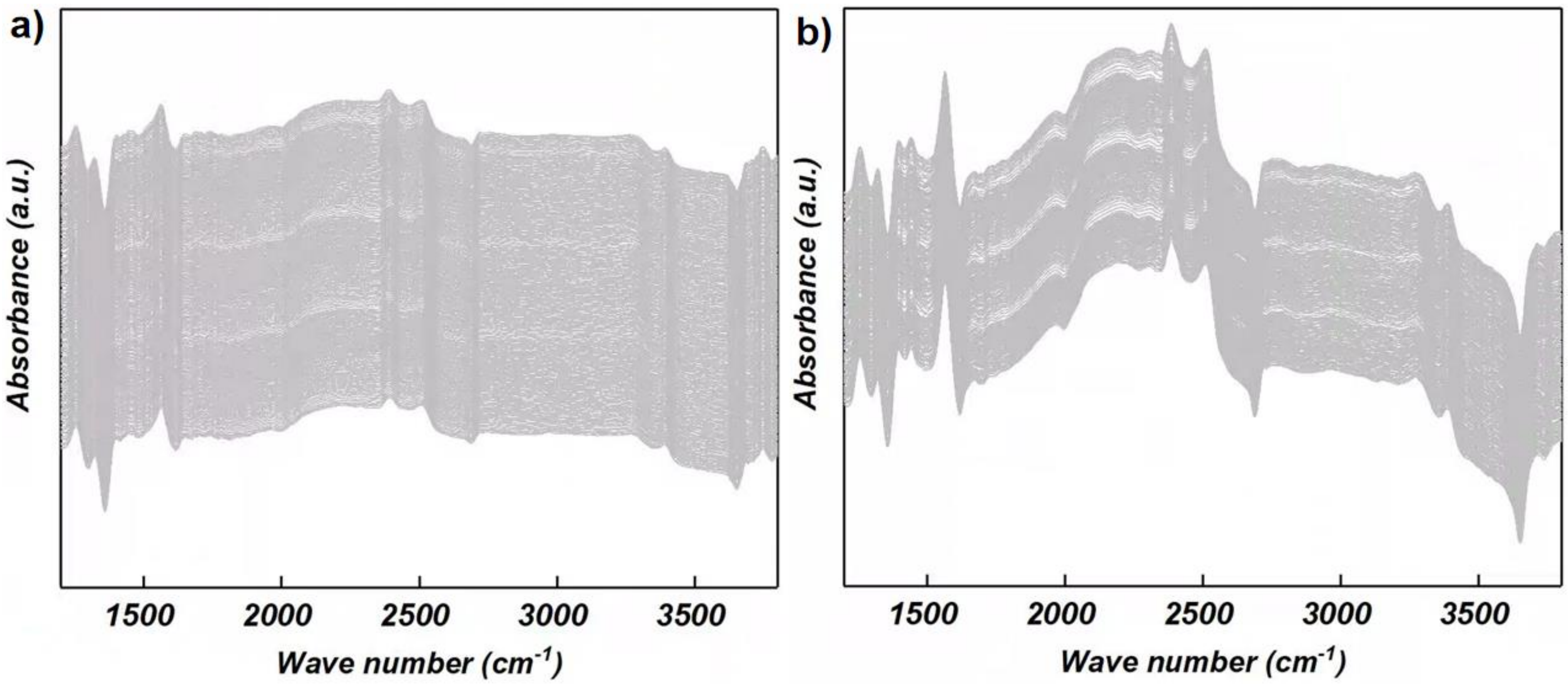

Figure S22 Time-resolved DRIFTS spectra at the wave number range of 1200-3800 cm^{-1} of a) OR and b) PL during NO+O_2/ND_3+O_2 modulation experiment at 150℃.

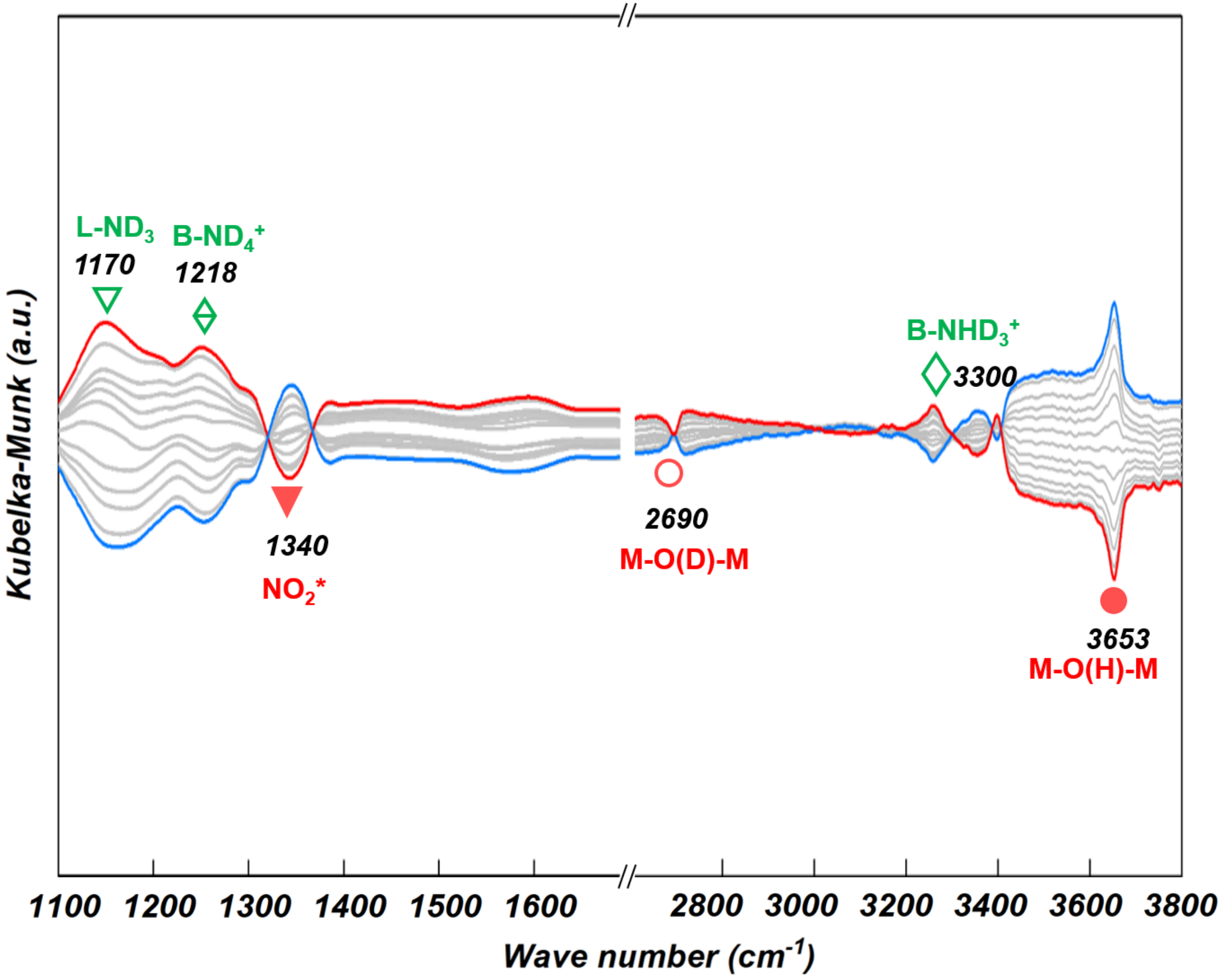

Figure S23 MES DRIFTS spectra of OR catalyst during $NO+O_2/ND_3+O_2$ modulation experiment at 150°C. Red colour spectra represent the phase when NH_3 was introduced, while blue colour represents the phase when NO was introduced.

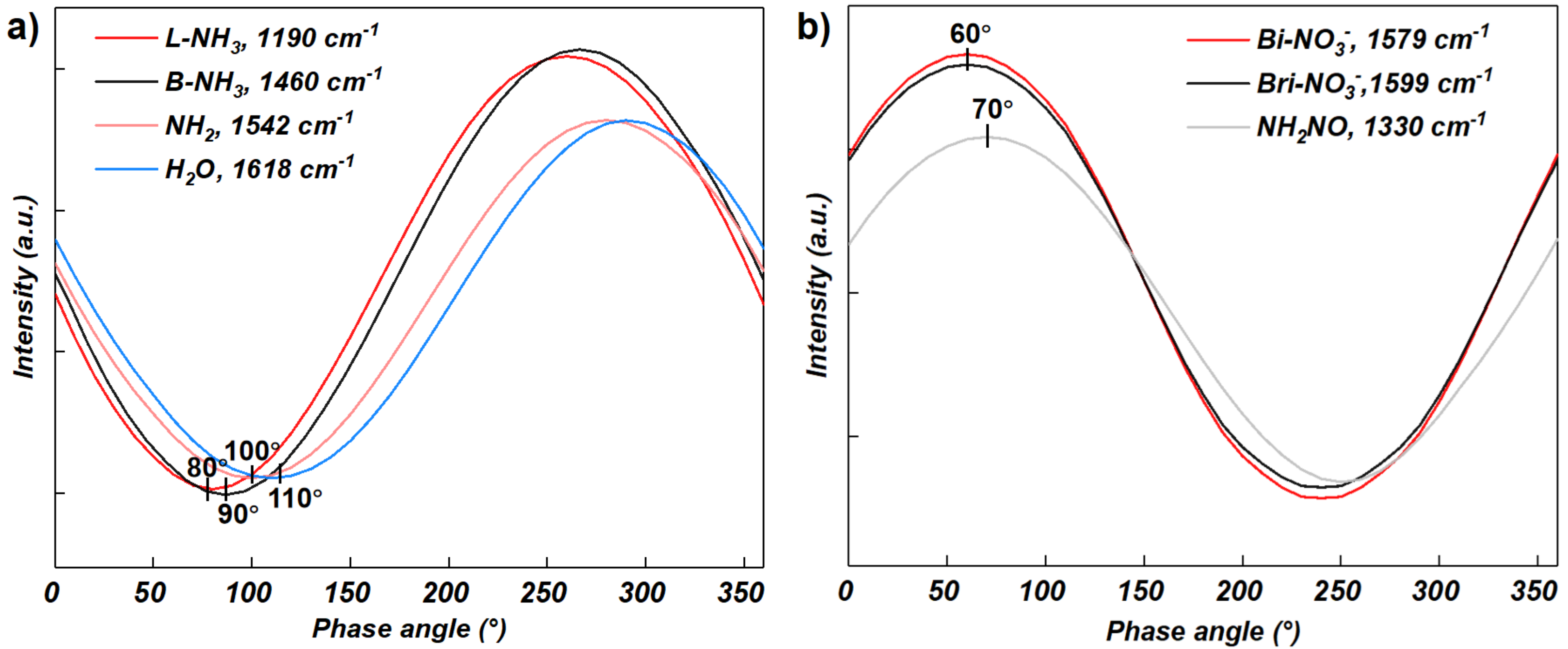

Figure S24 Phase angle dependence of FTIR signal intensity of (a) L-NH_3, B-NH_3, NH_2, H_2O, and (b) Bi-NO_3^-, Bri-NO_3^-, NH_2NO during NO+O_2/ND_3+O_2 modulation experiment of PL catalyst at 150°C. The phases of L-NH_3, B-NH_3, NH_2, H_2O were at 80°, 90°, 100°, and 110°, respectively. The phases of Bi-NO_3^-, Bri-NO_3^- were at 60°, NH_2NO was at 70°.

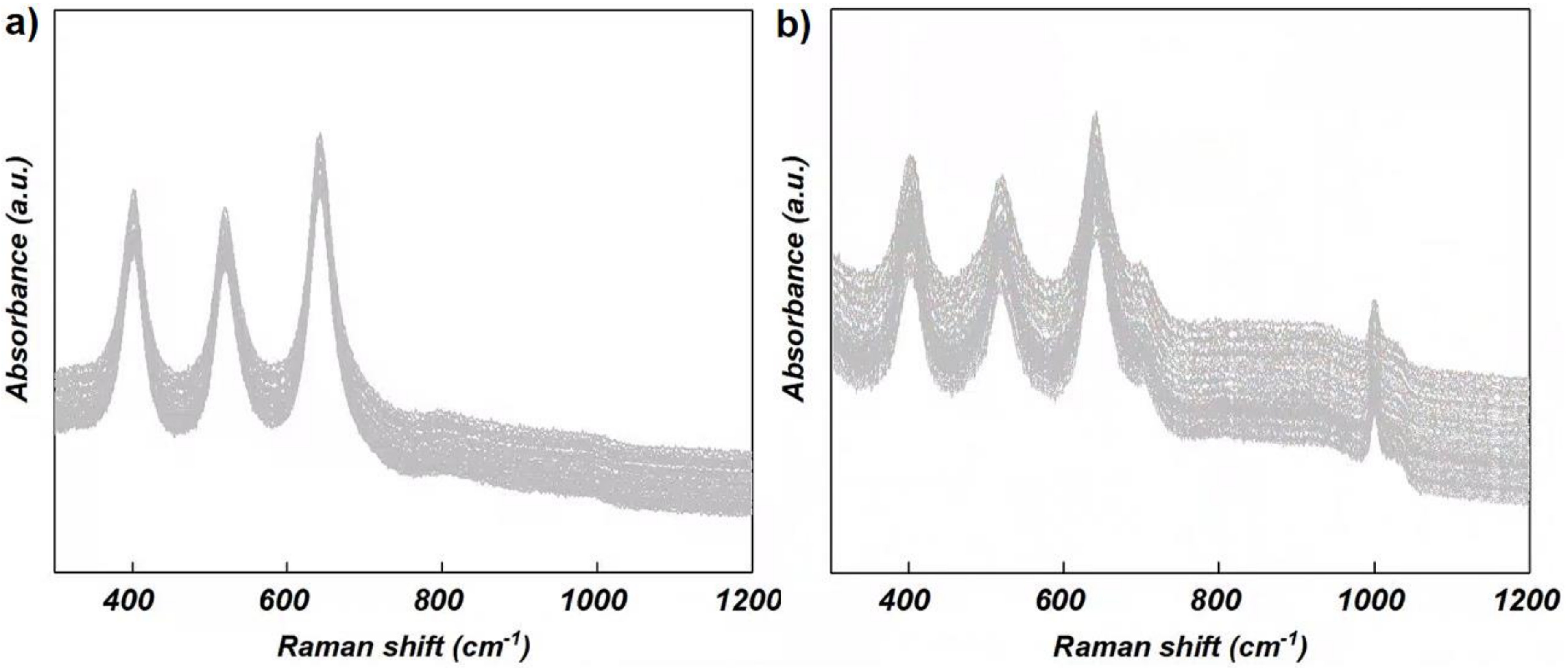

Figure S25 Time-resolved Raman spectra at the Raman shift range of 300-1200 cm^{-1} of a) OR and b) PL during $NO+O_2/NH_3+O_2$ modulation experiment at 150℃.

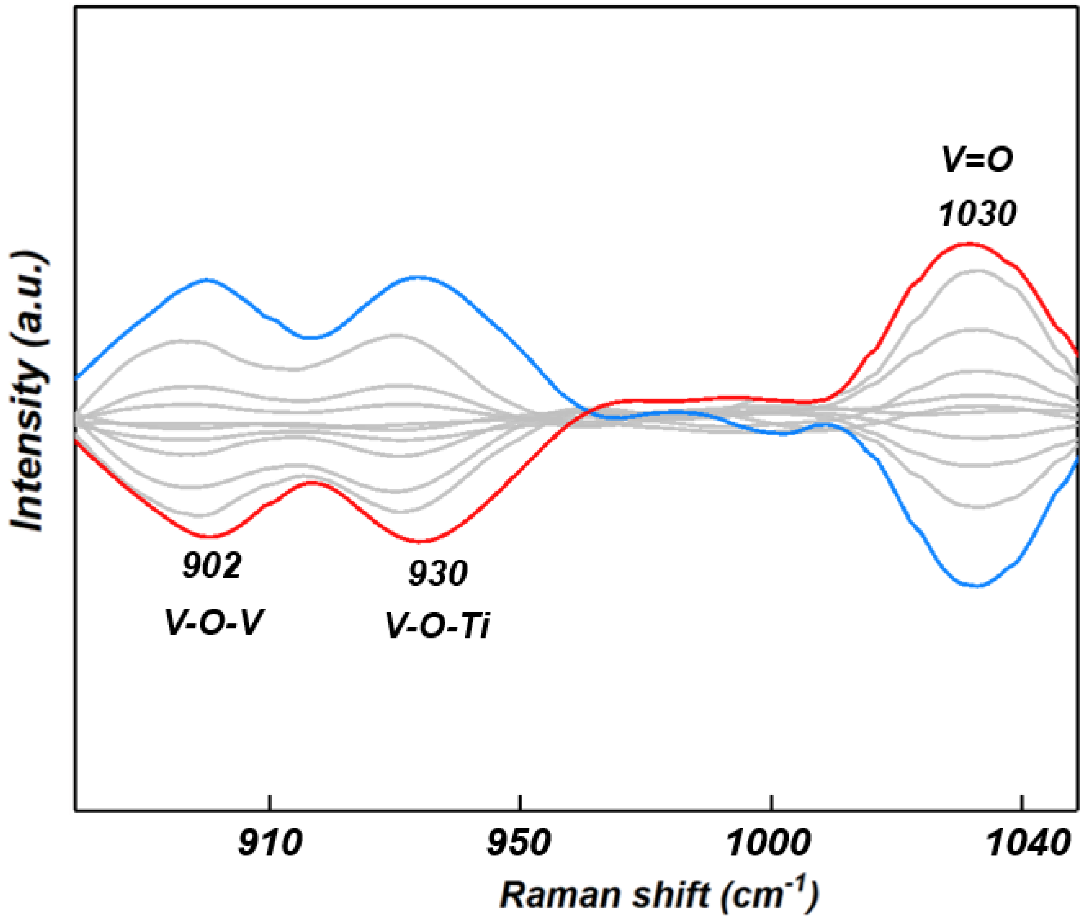

Figure S26 MES Raman spectra of VO_x/TiO_2 PL during $NO+O_2/NH_3+O_2$ modulation experiment at 150℃.

Instruction: Phase resolved Raman experiments were conducted on the VO_x/TiO_2 (PL) catalyst, revealing response peaks at 902, 930, and 1030 cm^{-1} during the transition of the reaction atmosphere. This substantiates that the peaks observed at 902 and 930 cm^{-1} are ascribed to two inequivalent V-O-Ti sites. These results provide further verification that the presence of vanadia is responsible for the observed surface reactant species.

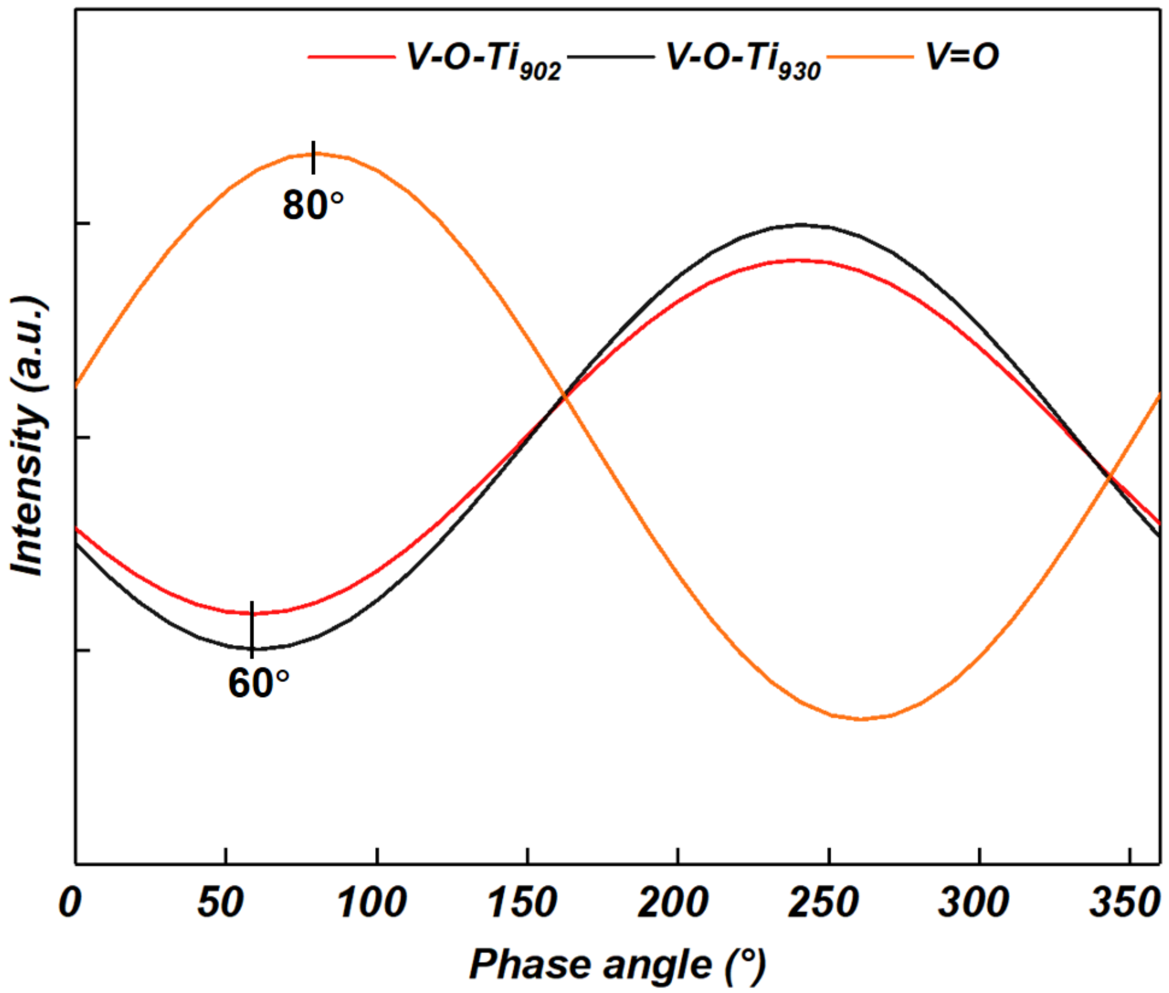

Figure S27 Phase angle dependence of Raman signal intensity of V-O-Ti$_{902}$, V-O-Ti$_{930}$ and V=O during NO+O_2/ND_3+O_2 modulation experiment of PL catalyst at 150℃. V-O-Ti$_{902}$ and V-O-Ti$_{930}$ represent Raman vibrational peaks at 902 cm^{-1} and 930 cm^{-1}, respectively.

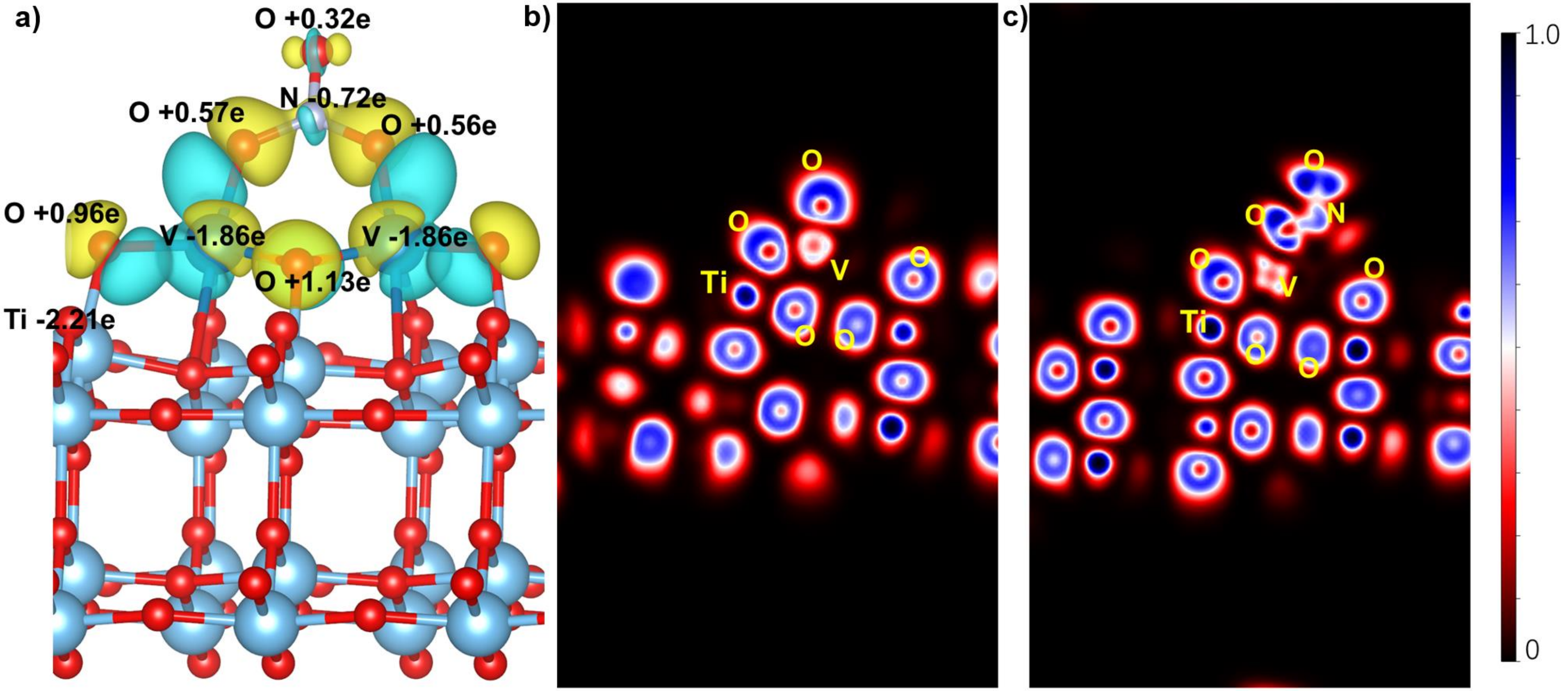

Figure S28 (a) Three-dimensional visualization of charge density difference of VVTi-NO with the isosurface of 0.057 e/Å. Bader charges are labelled on selected atoms. (b,c) Two-dimensional visualization of electron localization function (ELF) of VVTi before and after NO adsorption

Instruction: Bader charge analysis was employed to investigate the impact of NO introduction on the V-O bond. It revealed that after the introduction of NO, there was a higher concentration of charge near NO, while the charge distribution around V atoms weakened, suggesting a decrease in the covalence of V-O bonds.

7 Comparison of Observed Vibrational Modes and Their Assignments During MES of NH_3-SCR over VO_x-WO_x/TiO_2

The role of isotopically labelled reactant ND_3:

IR peaks associated with NH_3 shift to lower wavenumbers due to the spectral isotope effect of ND_3. Notably, differences in the isotope nuclear mass result in different vibrational frequencies, causing shifts in the infrared spectrum. [8] From the harmonic oscillator model, the relationship between vibrational frequencies and mass and force constant is as below:

$$v = \frac{1}{2\pi}\sqrt{\frac{K}{M}}$$

K is the force constant

For diatomic molecules (m_1, m_2), $M = \frac{m1m2}{m1+m2}$

$$v_{N-H} = \frac{1}{2\pi}\sqrt{\frac{K}{M(N-H)}}$$

$$v_{N-D} = \frac{1}{2\pi}\sqrt{\frac{K}{M(N-D)}}$$

$$\frac{v_{N-H}}{v_{N-D}} = \sqrt{\frac{M(N-D)}{M(N-H)}} = 1.37$$

Marberger *et al.* identified NH_3* at 1603 cm^{-1} using MES-FTIR.[4] After employing ND_3 as the reactant, the adsorption peak of ND_3* shifted to 1170 cm^{-1}. Additionally, the position of D_2O shifted from the original H_2O infrared peak at 1618 to 1385 cm^{-1}. However, the infrared absorption peak positions of bidentate nitrate and bridging nitrate remained at 1579 and 1599 cm^{-1}. Therefore, by using isotopically labeled reactant ND_3, the influence of adsorbed NH_3 and H_2O infrared peaks was excluded, enabling the accurate identification of nitrate species.

Comparison of Observed Vibrational Modes and Their Assignments During MES of NH_3-SCR over VO_x-WO_x/TiO_2

The vibrational modes observed by Marberger during transient SCR catalysis over a VO_x-WO_x/TiO_2 are compared with this work in Table S4. Discrepancies are highlighted in bold. A key discrepancy is the assignment of the 1282 cm^{-1} mode observed by Marberger *et al.* [8] to NH_3^*. By contrast, we observed the v_s(N-H) mode of NH_3^* at 1190 cm^{-1}, which was confirmed by isotopic substitution with ND_3 and the v_s(N-D) mode was observed at 1170 cm^{-1}. The calculated isotopic shift factor for substitution of ^{14}N-H with ^{14}N-D is 1.3685. Therefore, the expected v_s(N-D) mode is expected at 870 cm^{-1}. However, the v_4 mode for ND_3 gas is observed at 1191 cm^{-1} (commensurate NH_3 gas mode at 1627 cm^{-1}) [18]. Thus, the assigned species appears to be correct in this work. The 1282 cm^{-1} assignment by Marberger *et al.* is also not consistent with the 1250 cm^{-1} assignment for NH_3^* observed during transient conditions by Marberger *et al.* (highlighted in blue). Altogether, these discrepancies weigh against Marberger *et al.* assignment of 1282 cm^{-1} to NH_3^* and in support of our assignment of bridged NO_3^-.

Next, the commensurate assignment at ~1600 cm^{-1} to NH_3^* by Marberger *et al.* vs. bridged NO_3^- is in question. Based on the 1627/1191 = 1.366 isotopic shift factor for the ν_4 mode of NH_3/ND_3 gas [18], the v_4 mode of adsorbed NH_3^* is expected at 1170*1.366 = 1598 cm^{-1}. This is consistent with Marberger *et al.* assignment of NH_3^* and not bridged NO_3^-. However, the same 1599 cm^{-1} band is observed during ND_3 flow. This would be consistent with a bridged NO_3^- species lacking any hydrogen atoms. Thus, the assignment for 1599 cm^{-1} appears contradictory.

It is further noted that in the MES-PSD spectra in this work, the band at 1618 cm^{-1} assigned to H_2O^* evolves with phase angle such that the shape of the band changes asymmetrically. This is contrast to the band at 1385 cm^{-1} for D_2O^*, whose peak position is constant with changing phase angle. This indicates two overlapping bands in the envelope of the ~1620 cm^{-1} band. It is proposed herein that the second band arises from the v_4 mode of NH_3^*.

The last discrepancy is the assignment of 1333 and 1340 cm^{-1} to ν(S=O) and cis-$N_2O_2^-$, respectively. It is plausible that NH_3 gas reversibly adsorbs at sulfate sites present on the catalyst studied by Marberger *et al.* However, such sulfate sites are absent in the catalyst studied in this work. It is therefore proposed that cis-$N_2O_2^-$ might be present on the catalyst studied by Marberger *et al.*, but its observation is obscured by coordination to sulfate sites.

In summary, by careful application of isotopes studied by vibrational spectroscopy, we have correctly identified the key surface intermediates responsible for SCR catalysis. The key role for Lewis acid sites proposed by Marberger *et al.* hinges on their assignment of NH_3^*. In fact, this appears to be a bridged NO_3^-* species. Thus, all data appear consistent with a dual site reaction mechanism.

Table S4 Comparison of observed vibrational modes and assignments by Marberger et al. [8] and in this work. Not all vibrational modes from MES-PSD (Figure 4) were reported in the text by Marberger et al. Therefore, assigned modes in Figure 4 in [8] were extracted and are compared to those that were reported in the text [8].

Marberger assignment	Fig. 1 (transient): modes identified in text (Marberger)	Fig. 3 (MES-PSD): modes identified in text (Marberger)	Fig. 4 (MES-PSD): modes assigned and extracted from figure (by BMM) (Marberger)	Fig. 4 (MES-PSD): modes identified in text (Marberger)	Fig. 4 (MES-PSD): comparable identified modes (this work)	This work (MES-PSD) assignments
V-OH			3660		3653	
$[NH_4^+]^*$	3200-2700				3300	
NH_3^*	3250				3260	
V=O			2037	2045		
W=O			2002	2005		
$[NH_4^+]^*$	1670					
NH_3^*	1603		1599	1603	1599	Bri-NO_3^-
NH_2NO^*		1490	1489	1490		
$[NH_4^+]^*$			1460		1460	$[NH_4^+]$*
$[NH_4^+]^*$	1423					
		1400				
$[NH_4^+]^*$			1374		1370	$[NH_4^+]$*
S=O			1333		1340	cis-$N_2O_2^-$
NH_3^*			1282		1288	Bri-NO_3^-
NH_3^*	1250				1190	NH_3*

8 Investigation on Reaction Pathways and Rate-determining Steps

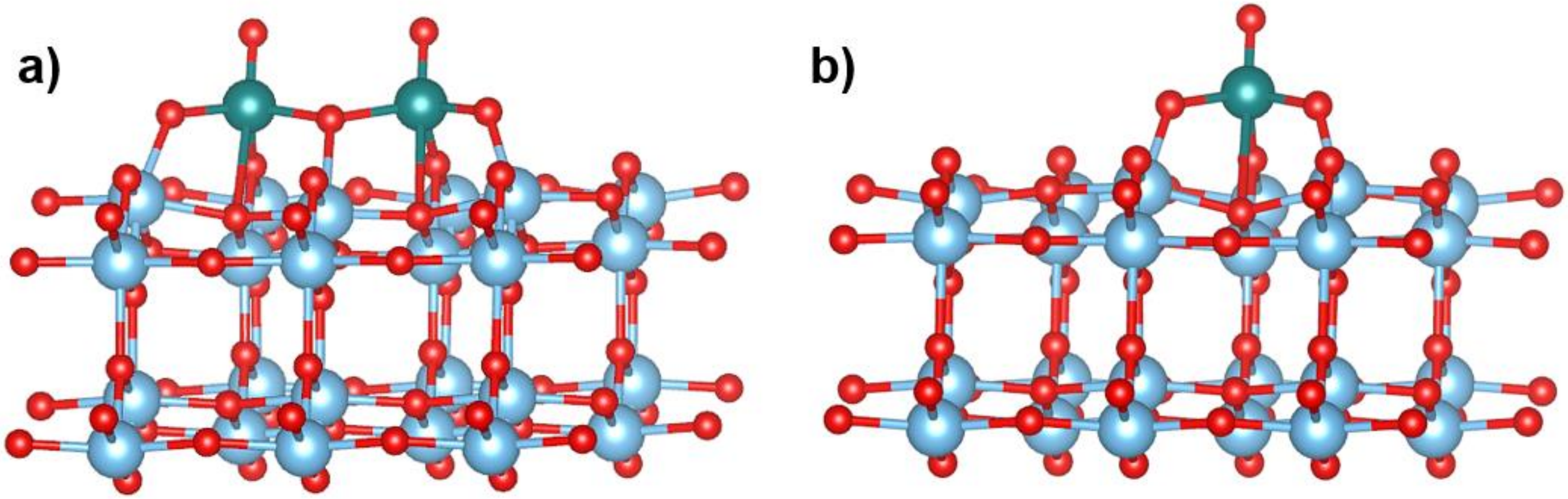

Figure S29 a) dimeric and b) monomeric mono-oxo vanadia/TiO_2 models used to calculate the catalytic reaction pathways. Red, cyan, green, blue, and white circles denote O, Ti, V atoms, respectively [15].

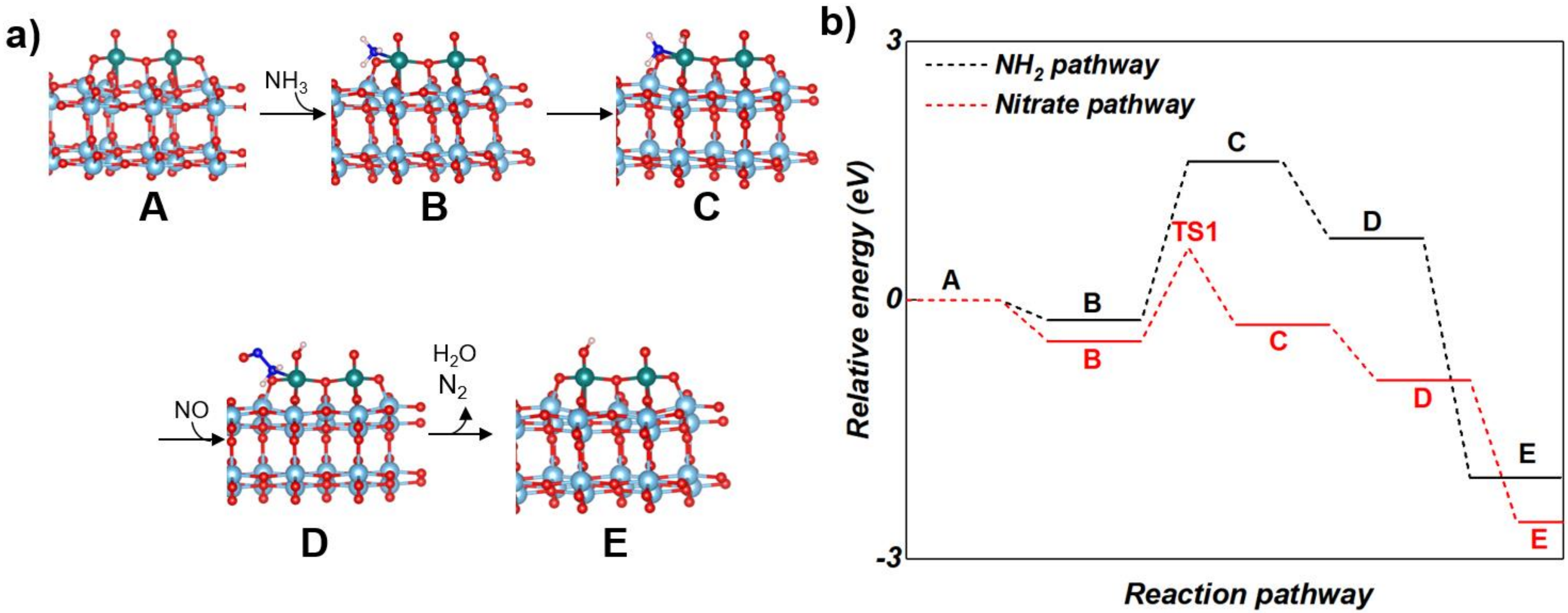

Figure S30 a) NH_2 reaction pathway on dimeric vanadia sites. b) Reaction energies of NH_2 pathway and nitrate pathway in the manuscript.

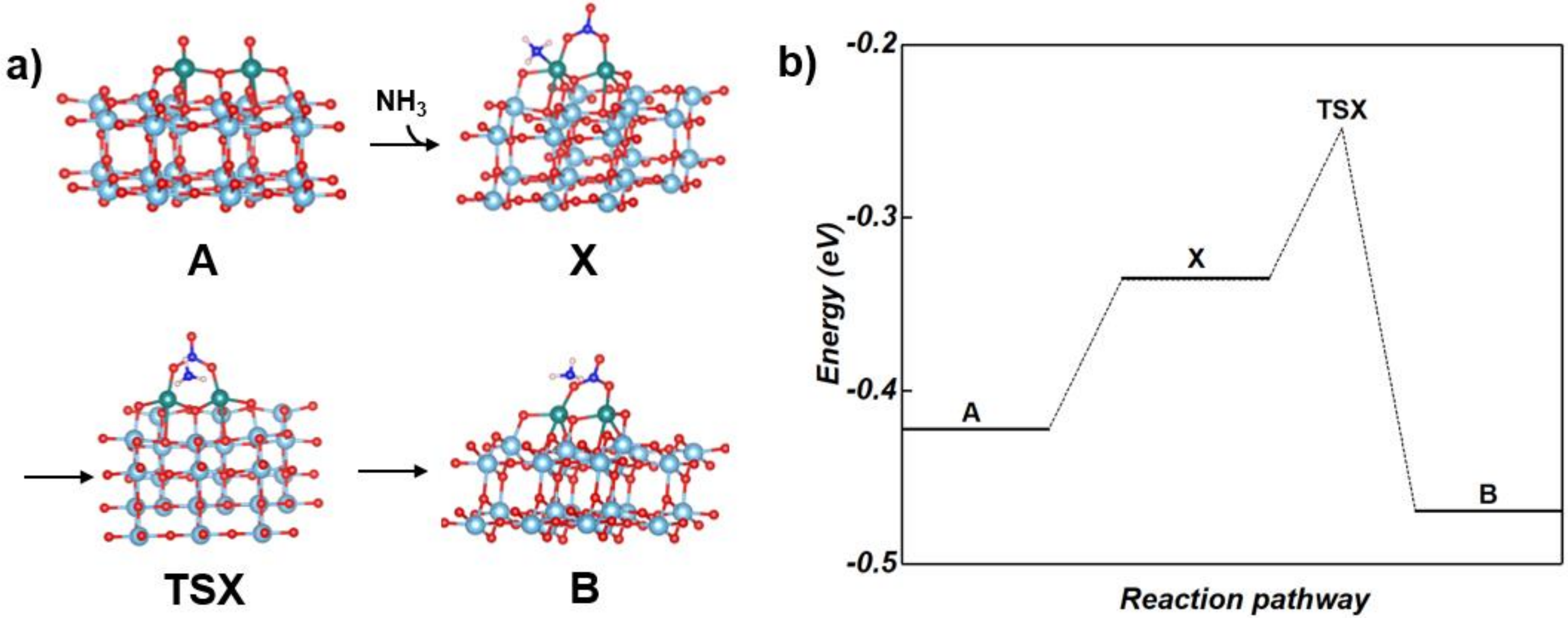

Figure S31 a) L-NH_3 pathway on dimeric vanadia sites and b) corresponding reaction energies. Configuration A represents dimeric vanadia sites, configuration X represents adsorbed L-NH_3 and nitrate, configuration TSX represents the transition state, and configuration B is identical to Fig 5a.

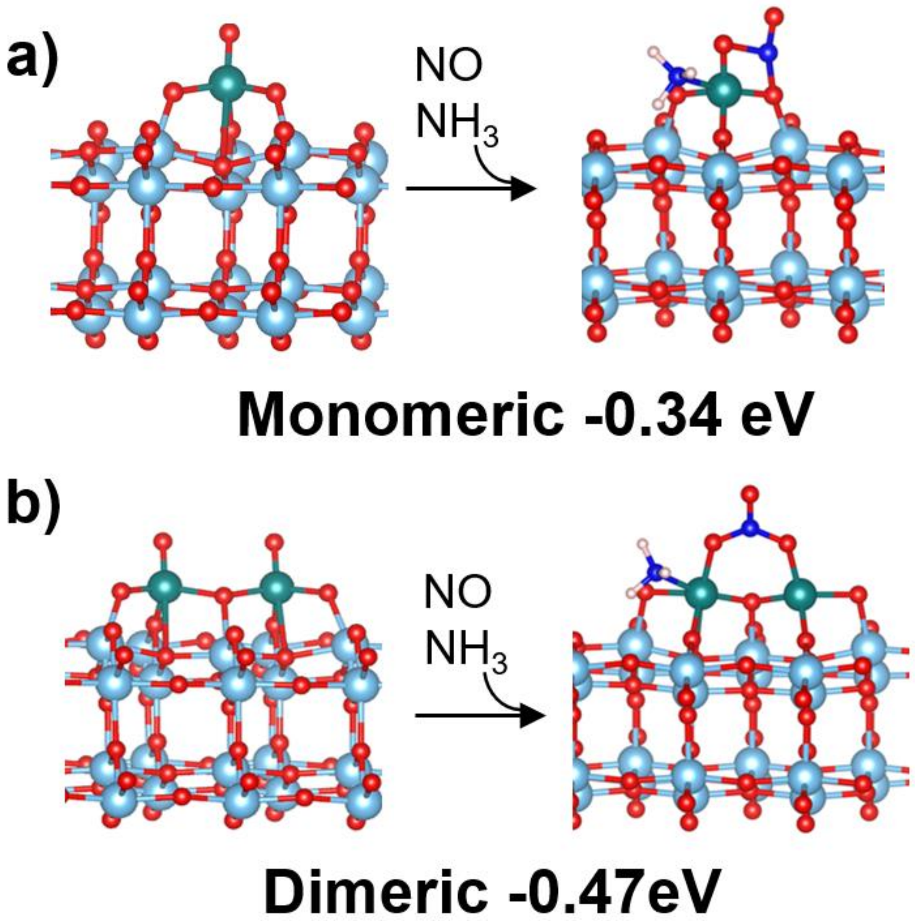

Figure S32 Nitrate formation energy on a) monomeric and b) dimeric vanadia sites. The formation energy of nitrate by NO adsorption on dimeric vanadia sites was -0.47 eV, which was lower than the -0.34 eV on monomeric vanadia sites.

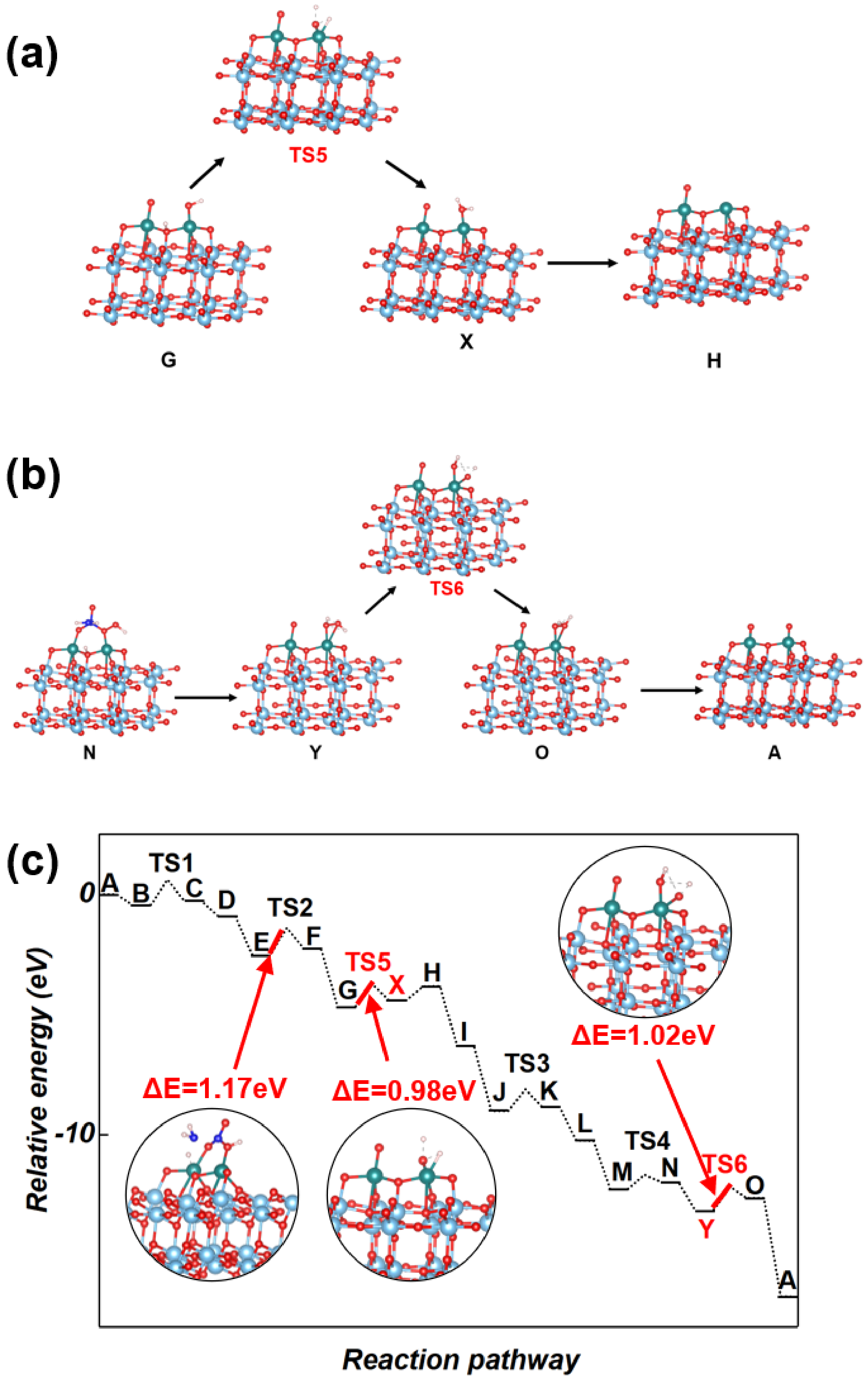

Figure S33 The transition states for the generation of H_2O for G→H (a), N→O (b) processes and their related positions in the whole reaction cycle (c) over the surfaces of a dimeric surface vanadia sites.

Table S5 Frequency for H_2O in transition states TS5 and TS6

Mode	Frequency/THz (TS5)	Frequency/THz (TS6)
**1**	39.3	47.4
**2**	30.92	36.6
**3**	24.5	35.1
**4**	22.1	33.4
**5**	14.7	29.4
**6**	8.34	14.1
**7**	3.41	10.9
**8**	1.73	2.8
**9**	-31.33	-4.6

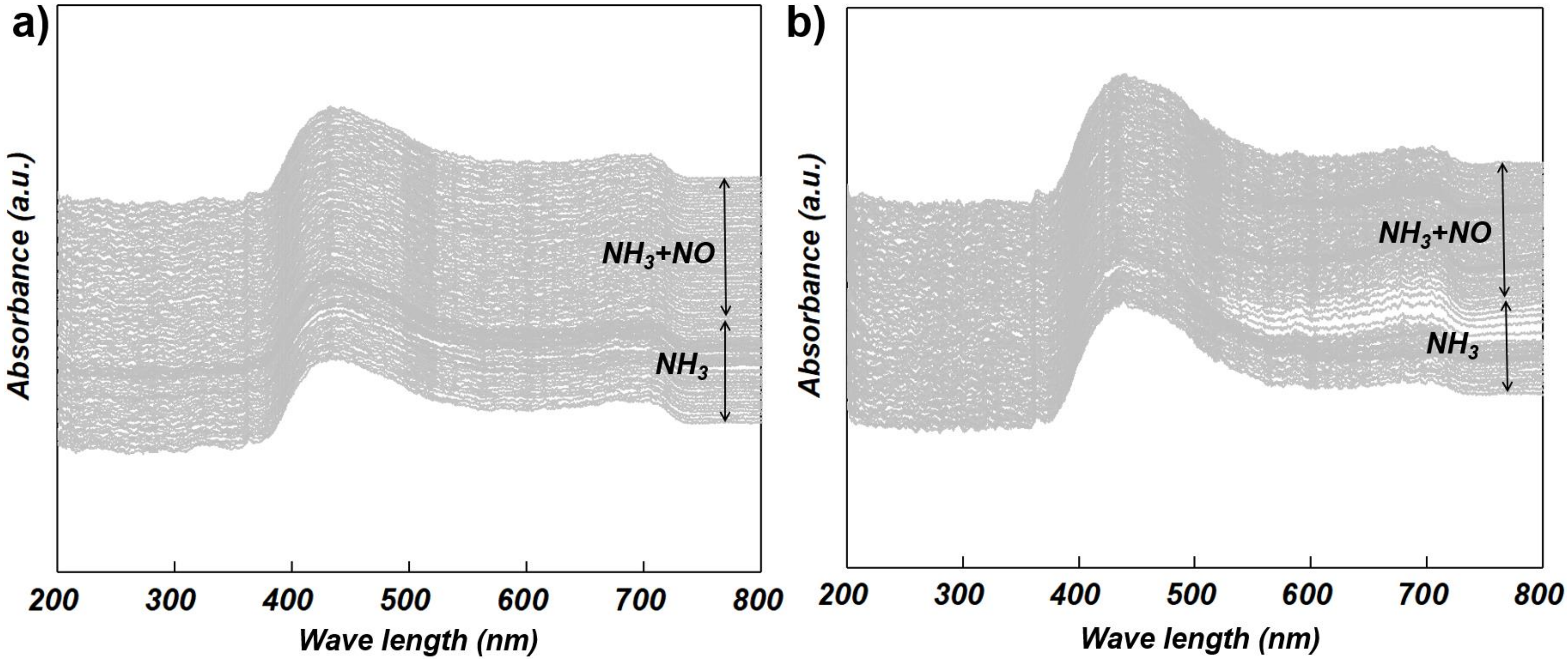

Figure S34 UV-vis spectra at the wave length range of 200-800 nm of a) OR and b) PL under NH_3 and NH_3+NO environment in sequence at 150 °C.

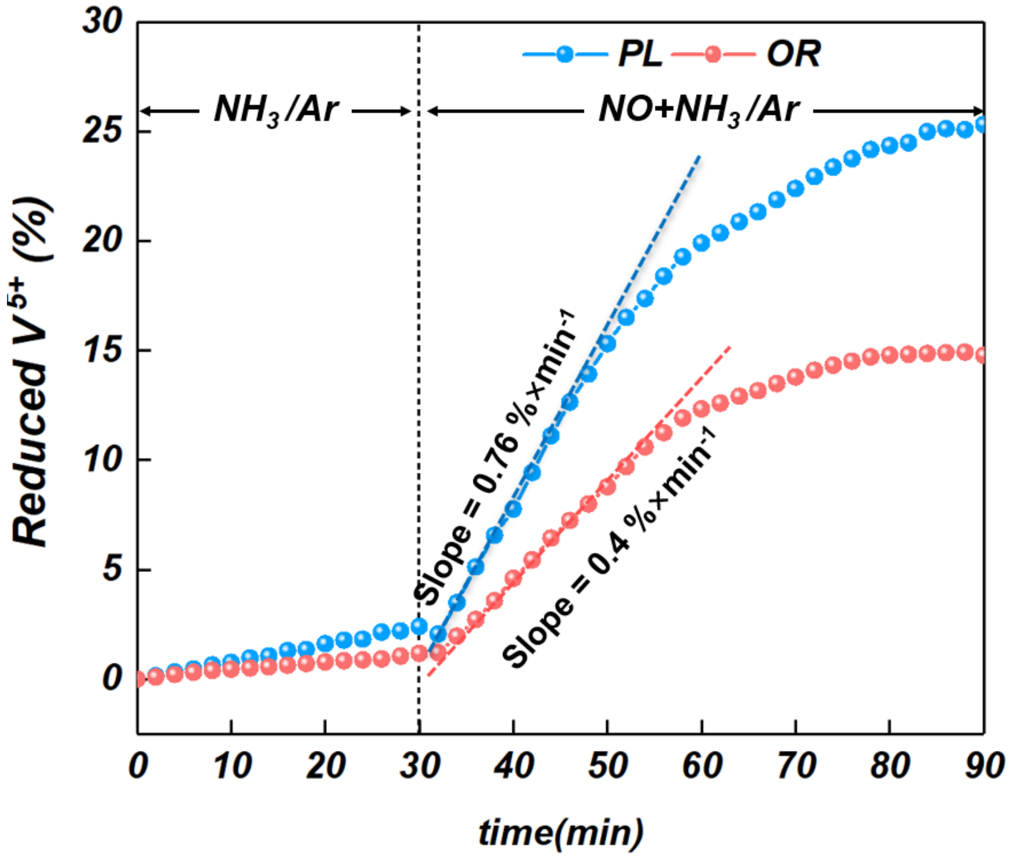

Figure S35 Reduction of the surface V^{5+} sites for OR and PL, as a function of environmental conditions. (2000 ppm of NH_3/Ar, 2000ppm of NO+2000ppm of NH_3/Ar in sequence). Fine catalyst powder was placed into the *in situ* reaction cell, pre-treated in a 10 % CO/Ar (50 ml/min) flow at 400 °C for 1h to yield 100% V^{4+}, and then pre-treated in 10 % O_2/Ar (50 ml/min) flow at 400 °C for 1h to yield 100% V^{5+}. Cooling the samples to the corresponding temperature, the catalyst was sequentially exposed to 2000ppm of NH_3/Ar (50 ml/min), and 2000ppm of NO+2000ppm of NH_3/Ar (50 ml/min). The UV-vis spectra were collected every 2min [16].

Table S6 Calculated rates of V^{5+} reduction steps from UV-vis spectroscopy in Figure S31.

	NO+NH_3 reduction step		
	V^{5+} (%)	rate (%/min)	the specific reduction rates (×10^{-2} min^{-1})
PL	97.9	0.76	0.78
OR	98.8	0.40	0.40

Instruction and Clarification: The *in situ* UV-vis time-resolved spectroscopy was carried out to explore the kinetics of the V^{5+} reduction step, which was determined as the rate-determining step (RDS) of the PL catalyst based on the finding from the DFT calculations (Table S4). The catalysts were pre-treated at 400 °C under 5% O_2/Ar to obtain 100% V^{5+}, and then exposed to NH_3 and NH_3 +NO in sequence with online UV-vis spectral collection. The complete reduction of the surface V^{5+} to V^{4+} sites were calibrated in flowing 10% CO/Ar at 400 °C for 5 h, which were quantified by normalization of the *d*−*d* transition band at 799 nm.

9 Investigation on the Factors Underlying the Diminishing Activity with Prolonged Plasma Treatment Time

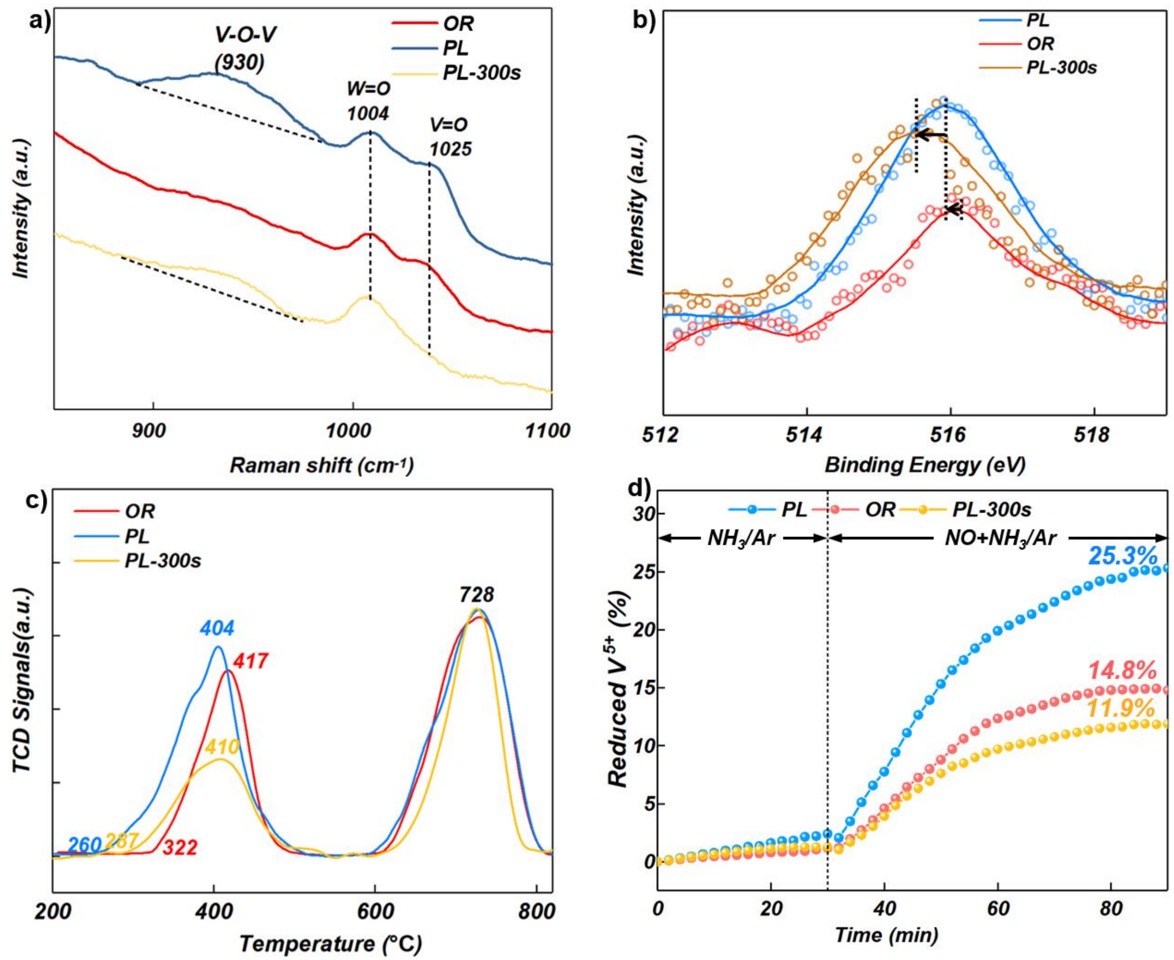

Figure 36 a) Raman spectra of OR, PL and PL-300s. **b)** V 2p spectra of OR, PL and PL-300s samples. **c)** H_2-TPR profiles of OR, PL and PL-300s samples. **d)** Reduction of the surface V^{5+} sites for OR, PL, PL-300s and PL-uncalcined samples as a function of environmental conditions. (2000 ppm of NH_3/Ar, 2000ppm of NO+2000ppm of NH_3/Ar in sequence).

Supplementary References

1. Yan, T. *et al.* Promoter rather than Inhibitor: Phosphorus incorporation accelerates the activity of V_2O_5–WO_3/TiO_2 catalyst for selective catalytic reduction of NO_x by NH_3. ACS Catal. **10**, 2747-2753 (2020).
2. Lai, J.-K. *et al.* Structure–activity relationships of hydrothermally aged titania-supported vanadium–tungsten oxide catalysts for SCR of NO_x emissions with NH_3. ACS Catal. **11**, *12096-12111* (2021).
3. Song, I. *et al.* Simple physical mixing of zeolite prevents sulfur deactivation of vanadia catalysts for NO_x removal. Nat. Commun. **12**, *901* (2021).
4. Dann, E. K. *et al.* Structural selectivity of supported Pd nanoparticles for catalytic NH_3 oxidation resolved using combined operando spectroscopy. Nat. Catal. 2, 157-163 (2019).
5. Zhang, Y. *et al.* Using transient FTIR spectroscopy to probe active sites and reaction intermediates for selective catalytic reduction of NO on Cu/SSZ-13 catalysts. ACS Catal. **9**, *6137-6145* (2019).
6. Nuguid, R. J. G. et al. Modulated excitation raman spectroscopy of V_2O_5/TiO_2: mechanistic insights into the selective catalytic reduction of NO with NH_3. ACS Catal. **9**, *6814-6820* (2019).
7. Ferri, D. *et al.* Revealing the dynamic structure of complex solid catalysts using modulated excitation X-ray diffraction. Angew. Chem. Int. Ed. Engl. **53**, *8890-8894*, (2014).
8. Marberger, A. et al. The significance of lewis acid sites for the selective catalytic reduction of nitric oxide on vanadium-based catalysts. Angew. Chem. Int. Ed. Engl. **55**, *11989-11994*, (2016).
9. Hwang, J. *et al.* Regulating oxygen activity of perovskites to promote NOx oxidation and reduction kinetics. Nat. Catal. **4**, *663-673* (2021).
10. Inomata, Y. *et al.* Bulk tungsten-substituted vanadium oxide for low-temperature NOx removal in the presence of water. Nat. Commun. **12**, *557* (2021).
11. Inomata, Y. *et al.* Bulk vanadium oxide versus conventional V_2O_5/TiO_2: NH_3–SCR catalysts working at a low temperature below 150 °C. ACS Catal. **9**, *9327-9331* (2019).
12. Wang, H. *et al.* Insight into the overlooked photochemical decomposition of atmospheric surface nitrates triggered by visible light. Angew. Chem. Int. Ed. Engl. **61**, *e202209201*, (2022).
13. Chen, S. *et al.* Coverage-dependent behaviors of vanadium oxides for chemical looping oxidative dehydrogenation. Angew. Chem. Int. Ed. Engl. **59**, *22072-22079*, (2020).
14. Xingtao G. *et al.* Investigation of surface structures of supported vanadium oxide catalysts by UV-vis-NIR diffuse reflectance spectroscopy. J. Phys. Chem. B. **104,** *1261-1268* (2000).
15. Guangzhi H. *et al.* Polymeric vanadyl species determine the low-temperature activity of V-based catalysts for the SCR of NO_x with NH_3. Sci. Adv. **4**, *eaau4637* (2018).
16. Zhu, M. *et al*. Reaction pathways and kinetics for selective catalytic reduction (SCR) of acidic NOx emissions from power plants with NH_3. ACS Catal. **7**, *8358-8361* (2017).
17. Hadjiivanov, K. *et al.* Isotopes in the FTIR investigations of solid surfaces. Spectrosc. Prop. Inorg. Organomet. Compd. **45**: *43-78* (2014).
18. Koops, Th. *et al*. Measurement and Interpretation of the Absolute Infrared Intensities of NH_3 and ND_3. J. Mol. Struct. **96**, *203-218* (1983).
